# Polymorphisms in DNA repair and oxidative stress genes associated with pre-treatment cognitive function in breast cancer survivors: an exploratory study

**DOI:** 10.1186/s40064-016-2061-4

**Published:** 2016-04-09

**Authors:** Theresa A. Koleck, Catherine M. Bender, Susan M. Sereika, Adam M. Brufsky, Barry C. Lembersky, Priscilla F. McAuliffe, Shannon L. Puhalla, Priya Rastogi, Yvette P. Conley

**Affiliations:** School of Nursing, University of Pittsburgh, 3500 Victoria Street, Pittsburgh, PA 15261 USA; Department of Biostatistics, Graduate School of Public Health, University of Pittsburgh, 130 De Soto Street, Pittsburgh, PA 15261 USA; Department of Epidemiology, Graduate School of Public Health, University of Pittsburgh, 130 De Soto Street, Pittsburgh, PA 15261 USA; Division of Hematology/Oncology, Magee-Womens Hospital of University of Pittsburgh Medical Center (UPMC), 300 Halket Street, Pittsburgh, PA 15213 USA; University of Pittsburgh Cancer Institute, 5150 Centre Avenue, Pittsburgh, PA 15232 USA; School of Medicine, University of Pittsburgh, 3550 Terrace Street, Pittsburgh, PA 15261 USA; Division of Breast Surgical Oncology, Magee-Womens Hospital of University of Pittsburgh Medical Center (UPMC), 300 Halket Street, Pittsburgh, PA 15213 USA; Department of Human Genetics, Graduate School of Public Health, University of Pittsburgh, 130 De Soto Street, Pittsburgh, PA 15261 USA

**Keywords:** Breast neoplasms, Cognition, Genetics, Polymorphisms, Oxidative stress, DNA repair

## Abstract

**Purpose:**

The purpose of this exploratory candidate gene association study was to examine relationships between polymorphisms in oxidative stress and DNA repair genes and pre-adjuvant therapy cognitive function (CF) in postmenopausal women diagnosed with early stage-breast cancer.

**Methods:**

Using a neuropsychological test battery, CF was assessed in 138 women diagnosed with breast cancer prior to initiation of adjuvant therapy and 81 age- and education-matched controls and summarized across eight composites. Participants were genotyped for 39 functional or tagging single nucleotide polymorphisms (SNPs) of select oxidative stress (*CAT*, *GPX1*, *SEPP1*, *SOD1*, and *SOD2*) and DNA repair (*ERCC2*, *ERCC3*, *ERCC5*, and *PARP1*) genes. Multiple linear regression was used to determine if the presence or absence of one or more minor alleles account for variability in CF composite scores. Based on regression findings from the analysis of individual SNPs, weighted multi-gene, multi-polymorphism genetic risk scores (GRSs) were calculated to evaluate the collective effect of possession of multiple protective and/or risk alleles.

**Results:**

Each CF composite was significantly (p < 0.05) associated with one or more oxidative stress and DNA repair gene polymorphisms evaluated either by SNP main effects and/or SNP-by-prescribed breast cancer treatment group interactions. Each computed GRS was found to be significantly (p < 0.001) related to its corresponding CF composite. All associations were positive suggesting that as overall genetic protection increases, CF composite score increases (indicating better performance).

**Conclusions:**

These findings suggest that genetic variation in the oxidative stress and DNA repair pathways may play an important role in pre-adjuvant therapy CF in breast cancer survivors.

**Electronic supplementary material:**

The online version of this article (doi:10.1186/s40064-016-2061-4) contains supplementary material, which is available to authorized users.

## Background

Pretreatment cognitive dysfunction has been well documented in women diagnosed with breast cancer (Wefel et al. 2008; Ahles et al. 2012); however, the mechanisms underlying this phenomenon as well as the variability in the presence and severity of cognitive dysfunction experienced by breast cancer survivors remain largely unknown. One biologically plausible mechanism that may at least partially account for pretreatment cognitive dysfunction and the observed variability is variation in response to oxidative stress and DNA damage (Janelsins et al. 2012; Ahles and Saykin 2007; Vardy et al. 2008). Evidence continues to build that supports the role of increased oxidative stress, insufficient antioxidant mechanisms, and/or deficient response to DNA damage in brain aging and cognitive decline (Coppedè and Migliore 2010; Jeppesen et al. 2012; Lillenes et al. 2011). Furthermore, oxidative damage and diminished DNA repair capacity have been implicated in more extreme cognitive dysfunction phenotypes, including mild cognitive impairment and Alzheimer’s disease (Bucholtz and Demuth 2013; Migliore et al. 2005; Jones et al. 1989).

The systemic environment and tumor microenvironment of a woman with breast cancer are characterized by increased, yet variable, levels of oxidative stress and DNA damage, with oxidative stress and subsequent DNA damage promoting breast cancer development and progression (Kang 2002; Jezierska-Drutel et al. 2013; Nourazarian et al. 2014). In one study of altered oxidative stress levels and breast cancer, Herrera et al. (2014) found evidence to support enhanced oxidative stress and reduced antioxidant defenses in plasma of postmenopausal women with primary ductal carcinomas of the breast at diagnosis compared to women 6 months post tumor removal and to healthy controls. Wang et al. also found increased levels of lipid peroxidation in breast cancer tissue but, in contrast to the previous study, upregulated antioxidant levels compared to tissue from healthy controls (Wang et al. 2014). In addition to being altered, research suggests that oxidative stress profiles are heterogeneous, differing between early and advanced stage breast cancers (Panis et al. 2012) and varying by tumor size and lymph node involvement (Saintot et al. 2002). In terms of DNA damage, chemotherapy naïve postmenopausal women with primary invasive ductal breast cancer were found to have higher basal levels of DNA damage and decreased DNA repair efficacy of peripheral blood lymphocytes (PBLs) compared to age-matched healthy women (Blasiak et al. 2004). Sanchez-Suarez et al. (2008) presented congruent findings: an assessment of PBLs from women with Stage 2 ductal carcinoma of the breast displayed higher DNA damage prior to initiation of adjuvant chemotherapy compared to age-matched healthy controls. Similar results were reported in a study of DNA damage and repair in PBLs in a heterogeneous sample of individuals (ages 1–59 years) with various cancer diagnoses compared to healthy controls (ages 22–50 years) with cells from cancer patients demonstrating higher levels of basal DNA damage. Considerable individual variation was also noted (Nadin et al. 2006).

The reported variability in oxidative stress and DNA damage profiles warrants investigation of genetic variation to account for differences in cognitive phenotypes of women diagnosed with breast cancer. Considering how increased oxidative stress and decreased DNA repair capacity impacts more extreme cognitive phenotypes as well as the vulnerability of the brain within the context of increased oxidative stress due to breast cancer, we hypothesize that variability in protection from oxidative damage and capacity to repair DNA may inform variability in the extent of cognitive dysfunction among breast cancer survivors. Thus, the purpose of this exploratory candidate gene association study was to examine relationships between variation in genes involved in oxidative stress (*CAT*, *GPX1*, *SEPP1*, *SOD1*, and *SOD2*) and DNA repair (*ERCC2*, *ERCC3*, *ERCC5*, and *PARP1*) and pre-adjuvant therapy CF in postmenopausal women with early stage breast cancer. Furthermore, cumulative multi-gene, multi-polymorphism genetic risk scores (GRSs) were calculated to evaluate the collective effect of possessing multiple significant polymorphisms.

## Methods

### Study participants

Participants in this candidate gene association study were recruited from a larger parent study investigating the effect of the adjuvant aromatase inhibitor therapy, anastrozole, on changes in CF in postmenopausal women with breast cancer (Bender et al. 2015). The sample (N = 219) was comprised of 138 women diagnosed with breast cancer and 81 age- and education-matched controls with no personal history of breast cancer. All participants were no greater than 75 years of age, able to speak and read English, completed at least 8 years of education, and had no previous history of cancer, psychiatric illness, or neurologic disease/trauma at time of enrollment into the parent study. In addition, women with breast cancer had a diagnosis of estrogen receptor positive, early-stage breast cancer (stages 1, 2, or 3a) based on the Tumor, Nodes, Metastasis Classification of Malignant Tumors with no clinical evidence of distant metastases (Edge et al. 2010). This study was approved by the University of Pittsburgh Institutional Review Board. Informed consent was obtained from all individual participants included in both the parent and genetic ancillary study.

### Evaluation of cognitive function

A battery of neuropsychological tests was used to assess cognitive function (CF). Women with cancer completed cognitive assessment after primary surgery but prior to initiation of adjuvant therapy. Control women completed the same cognitive assessment. The neuropsychological test battery was individually administered to study participants by trained research nurses. The selection of neuropsychological tests for the battery and reduction of individual neuropsychological test data into the eight following CF composites based on exploratory factor analysis have been described in detail previously (Bender et al. 2015):

1. Attention—Cambridge Neuropsychological Test Automated Battery (CANTAB) Rapid Visual Information Processing (Robbins et al. 1994)
2. Concentration—Digit Vigilance (Layfayette Clinical Insturments Company 1989)
3. Mental Flexibility—Delis Kaplan Executive Function System Color-Word Interference (Delis et al. 2001)
4. Executive Function—CANTAB Stockings of Cambridge (Robbins et al. 1994) and CANTAB Spatial Working Memory (Robbins et al. 1994)
5. Psychomotor Speed—Grooved Pegboard (Klove 1963) and Digit Symbol Substitution (Wechsler 1998)
6. Verbal Memory—Rey Auditory Verbal Learning (Rey 1964), Verbal Fluency Test, and Rivermead Story (Cockburn and Smith 1993)
7. Visual Memory—CANTAB Paired Associates Learning (Robbins et al. 1994) and Rey Complex Figure (Osterrieth 1944)
8. Visual Working Memory—CANTAB Stockings of Cambridge (Robbins et al. 1994) and Rey Complex Figure (Osterrieth 1944)

### Covariate assessment

In order to control for the potential effects of age, intelligence, mood, and pain on CF, age, estimated verbal intelligence (National Adult Reading Test-Revised) (Nelson 1981), and levels of depressive symptoms (Beck Depression Inventory-II) (Beck et al. 1996), anxiety (POMS tension-anxiety subscale) (McNair et al. 1992), fatigue (POMS fatigue-inertia subscale) (McNair et al. 1992), and pain (Brief Pain Inventory) (Cleeland 1989) were also assessed. All participants in this study had complete covariate/confounder information.

### SNP selection and genotyping

Functional polymorphisms for five candidate oxidative stress genes (Catalase, *CAT*; Glutathione Peroxidase 1, *GPX1*; Selenoprotein P, Plasma 1, *SEPP1*; Superoxide Dismutase 1, Soluble, *SOD1*; and Superoxide Dismutase 2, Mitochondrial, *SOD2*) and four candidate DNA repair genes (Excision Repair Cross-Complementation Group 2, *ERCC2*; Excision Repair Cross-Complementation Group 3, *ERCC3*; Excision Repair Cross-Complementation Group 5, *ERCC5*; and Poly (ADP-ribose) Polymerase 1, *PARP1*) were identified from the literature (Hamanishi et al. 2004; Valenti et al. 2004; Islam et al. 2007; Jiang et al. 2001; De Haan et al. 1998; Meplan et al. 2007; Spencer et al. 2008; Hooker et al. 2007; Mizutani 2007; Lockett et al. 2004). When a functional polymorphism was not identified or did not fully represent all of the variability in the gene, tagging SNPs were selected using the Phase III HapMap database. Criteria for selecting tagging SNPs included: R^2^ of ≥0.8; minor allele frequency ≥20 %; and selected for Caucasian and African ancestry, which represents parent study subjects. In total, 39 functional or tagging SNPs were selected for evaluation (Table 1).

**Table 1:** Candidate DNA repair and oxidative stress genes and associated SNPs

DNA repair genes		Oxidative stress genes	
*ERCC2*	*ERCC5*	*CAT*	*SEPP1*
rs13181	rs11069498	rs1001179^a^	rs230819
rs1799786	rs2296147	rs10488736	rs28919892
rs1799787	rs2296148^a^	rs2179625	rs3877899^a^
rs238406	rs4150355	rs511895	
rs238416	rs4150360	rs525938	
rs3916874	rs4771436	rs566979	*SOD1*
rs50871	rs751402	rs769214^a^	rs1041740
rs50872	rs873601		
*ERCC3*	*PARP1*	*GPX1*	*SOD2*
rs2134794	rs1136410^a^	rs1050450^a^	rs4880^a^
rs4150402	rs2271347		rs5746136
rs4150407	rs3219058		rs8031
rs4150477	rs3219090		

*SNP* single nucleotide polymorphism

^a^Functional polymorphism

Genetic samples were collected from June 2008 to May 2014. Three milliliters of whole blood or two milliliters of saliva were obtained for genotyping. DNA was extracted from PBLs using a simple salting out procedure or from saliva utilizing the protocol and reagents supplied with the Oragene DNA collection kits (DNA Genotek Inc 2012; Miller et al. 1988). Genotypes were determined using either an iPLEX MassARRAY multiplex assay platform (Sequenom, San Diego, CA) or a TaqMan allele discrimination platform (Thermo Fisher Scientific Inc., Waltham, MA). Genotypes were double called by individuals blinded to subject phenotypes and discrepancies addressed by reviewing raw data or re-genotyping. Participant genotypes were classified for data analysis based on the presence (i.e., homozygous variant genotype plus heterozygous genotype) or absence (i.e., wildtype genotype) of the minor allele.

### Statistical analysis

Analyses were performed using IBM^®^ SPSS^®^ Statistics Version 23 (IBM Corp., Armonk, NY). A detailed descriptive analysis of all data was first performed to identify any anomalies prior to modeling. Each SNP was tested for Hardy–Weinberg equilibrium using a Chi square goodness-of-fit test. To account for the heterogeneity of breast cancer tumors, women diagnosed with breast cancer were further classified prior to analysis using prescribed treatment regimen as a surrogate for disease characteristics, such as disease stage and aggressiveness. Subsequently, the analysis featured two groups of women diagnosed with breast cancer, Group A (prescribed chemotherapy followed by anastrozole, n = 55) and Group B (prescribed anastrozole alone, n = 83), as well as the reference, healthy age- and education-matched control group (n = 81). Hierarchical multiple linear regression modeling was employed to estimate relationships between individual SNPs and each CF composite score. Both main SNP effect only and SNP-by-group interaction models were fitted. In all models, the prescribed treatment groups, Group A and Group B, were compared to the reference, control group. Likewise, possession of one or more minor alleles (i.e., homozygous variant genotype plus heterozygous genotype) was compared to the reference, wildtype genotype. All models were adjusted for age, estimated verbal intelligence, and levels of depressive symptoms, anxiety, fatigue, and pain at study entry. Regression diagnostics were examined for each model. Potentially influential cases were identified and sensitivity analyses were conducted to evaluate the robustness of findings. In order to retain cases found to be influential due to extreme CF scores, scores were modified to be less extreme but still the highest/lowest CF score(s) for the affected composite. Unstandardized regression b-coefficients were obtained and tested at a two-tailed significance level of 0.05.

GRSs were then calculated for each participant to evaluate the collective effect of multiple DNA repair and oxidative stress polymorphisms on CF composite scores. Separate GRSs were calculated for each CF composite. SNP minor alleles found to be significantly (p < 0.05) negatively or positively associated with CF composites in the individual main effect only and/or interaction effect models were utilized in GRS calculations. In order to assign greater risk/protection to alleles with stronger associations, a weighted method was employed. Unstandardized regression b-coefficients from the individual SNP models were multiplied by 0 (absence) or 1 (presence) based on a participant’s genotype and prescribed treatment group membership and then summed. For example, the equation to calculate the verbal memory GRS would be as follows:

$$\begin{aligned}&{\text{Verbal Memory GRS }} = \left( {-.346*CAT{\text{rs}}566979 - {\text{G}}} \right)\\&\quad +\left({.282*CAT{\text{rs}}566979 - {\text{G }}*{\text{ GroupA}}}\right)+\left({.387*CAT{\text{rs}}566979 - {\text{G }}*{\text{GroupB}}}\right)\\&\quad+ \left( {-.129*ERCC5{\text{rs}}11069498 - {\text{G}}} \right) +\left({.536*ERCC5rs11069498 - {\text{G }}*{\text{ GroupA}}} \right)\\&\quad+\left({.190*ERCC5rs11069498 - {\text{G }}*{\text{ GroupB}}}\right) + \left( { - .075*ERCC5{\text{rs}}751402 -{\text{C}}} \right)\\&\quad + \left( {.486*ERCC5rs751402 - {\text{C}}*{\text{ GroupA}}} \right)+ \left( {.255*ERCC5{\text{rs}}751402 -{\text{C}}*{\text{ GroupB}}} \right)\\&\quad + \left({ -.074*ERCC5{\text{rs}}4150360 - {\text{T}}} \right) +\left({.568*ERCC5rs4150360 - {\text{T }}*{\text{ GroupA}}} \right)\\&\quad +\left({.104*ERCC5{\text{rs}}4150360 -{\text{T }}*{\text{ GroupB}}}\right)\end{aligned}$$

Thus, a participant prescribed chemotherapy plus anastrozole (Group A) who possessed the minor alleles for *CAT*rs566979 and *ERCC5*rs4150360 would have a verbal memory GRS of 0.43 calculated as follows:

$$\begin{aligned} & {\text{Verbal Memory GRS }} \hfill \\ & \quad= ( { - .346*1}) + ({.282*1*1}) + ({.387*1*0} ) +( { - .129*0}) + ({.536*0*1}) \hfill \\ & \qquad + ({.190*0*0}) + ({- .075*0}) +( {.486*0*1}) + ({.255*0*0}) + ({- .074*1} ) \hfill \\ & \qquad + ({.568*1*1} ) + ({.104*1*0}) = 0.43 \hfill \\ \end{aligned}$$

A lower GRS indicates greater genetic risk for poorer CF and a higher GRS indicates greater genetic protection. Please note that if influential observations were identified by the sensitivity analysis, b-coefficients from the models with modified extreme CF scores were used. The unique contributions of GRSs in explaining the variance in CF composite scores were evaluated in the final block in a hierarchical multiple linear regression model, adjusted for age, estimated verbal intelligence, and levels of depressive symptoms, anxiety, fatigue, and pain. Participants missing genetic data necessary for completion of a GRS calculation were not included in the GRS analysis.

## Results

Genotyping rates of the 39 SNPs ranged from 85.5 to 100 %. When considering the entire cohort (cases and controls), six SNPs were not in Hardy–Weinberg equilibrium: *ERCC2*rs1799786 (χ^2^ = 4.77, p = 0.029), *ERCC2*rs238416 (χ^2^ = 3.92, p = 0.048), *ERCC2*rs50871 (χ^2^ = 4.37, p = 0.037), *PARP1*rs1136410 (χ^2^ = 4.78, p = 0.029), *PARP1*rs3219090 (χ^2^ = 6.04, p = 0.014), and *SEPP1*rs28919892 (χ^2^ = 4.29, p = 0.038). Of these six SNPs, only one, ERCC2rs238416 (χ^2^ = 4.29, p = 0.038) was not in Hardy–Weinberg equilibrium when considering the control group alone. This deviation is most likely due to lack of random sampling from the population for both the cases and controls. Group-wise comparisons of participant characteristics revealed that study groups differed statistically, but not clinically significantly by age and estimated verbal intelligence (Table 2).

**Table 2:** Participant characteristics (N = 219)

Characteristic (measure)	Group A (n = 55) *Mean* *±* *SD* or n (%)	Group B (n = 83) *Mean* *±* *SD* or n (%)	Healthy controls (n = 81) *Mean* *±* *SD* or n (%)	F or χ^2^ test statistic p value
Age (years)	58.76 ± 5.47	62.47 ± 5.96	60.06 ± 6.08	<.001*
Education (years)	15.67 ± 2.78	14.95 ± 3.06	14.84 ± 2.91	.232
Estimated verbal intelligence (NART-R)	108.94 ± 8.87	107.04 ± 8.84	114.72 ± 7.84	<.001*
Depression (BDI-II)	5.24 ± 6.61	4.60 ± 4.65	4.83 ± 5.52	.760
Anxiety (POMS tension-anxiety subscale)	9.61 ± 6.14	6.97 ± 4.65	6.61 ± 5.63	.004*
Fatigue (POMS fatigue-inertia subscale)	5.11 ± 5.33	5.84 ± 6.35	5.74 ± 5.99	.759
Pain (BPI)	1.47 ± 1.96	1.55 ± 2.27	0.94 ± 2.07	.144
Marital status, married	38 (69.1)	54 (65.1)	46 (56.8)	.306
Number of children	1.75 ± 1.22	2.05 ± 1.39	2.12 ± 1.53	.283
Race, Caucasian	52 (94.5)	81 (97.6)	75 (92.6)	.337
Cancer stage				
Stage 1	25 (45.5)	69 (83.1)	–	–
Stage 2a	19 (34.5)	12 (14.5)	–	–
Stage 2b	5 (9.1)	2 (2.4)	–	–
Stage 3a	6 (10.9)	0 (0.0)	–	–

SD, standard deviation; Group A, prescribed chemotherapy plus anastrozole; Group B, prescribed anastrozole alone; NART-R, National Adult Reading Test-Revised; BDI-II, Beck Depression Inventory-II, POMS, Profile of Mood States; BPI, Brief Pain Inventory. One-way ANOVAs utilized to compare study cohort means of continuous variables. Pearson’s Chi square tests of independence used to examine the general associations between categorical variables

* p < .05

Results from the individual SNP variant regression analyses are reported in the table found in Additional file 1. Individual polymorphisms significantly (p < 0.05) associated with a particular CF composite by a SNP main effect and/or SNP-by-group interaction effect are listed in Table 3. A selection of results from the individual SNP analysis has been highlighted by CF composite in the text to follow; please note that all reported b-coefficients indicate the magnitude and direction of possession of one or more minor alleles on CF. For attention, possession of one or more *ERCC3*rs2134794 (b = −0.309, p = 0.010) or *ERCC5*rs873601 (b = −0.288, p = −0.015) minor alleles was associated with poorer performance regardless of group membership. SNP main effects also influenced mental flexibility, psychomotor speed, and concentration performance. For mental flexibility, a number of significant SNP main effects were observed over multiple oxidative stress and DNA repair genes: *ERCC2*rs13181 (b = −0.179, p = 0.031), *ERCC3*rs4150407 (b = 0.234, p = 0.016), *ERCC3*rs4150477 (b = 0.190, p = 0.038), *PARP1*rs2271347 (b = 0.202, p = 0.034), *SEPP1*rs230819 (b = 0.255, p = 0.018), and *SOD1*rs1041740 (b = 0.254, p = 0.006). Significant SNP main effects were also observed for psychomotor speed: *CAT*rs511895 (b = 0.237, p = 0.031), *CAT*rs769214 (b = −0.421, p = 0.020), *ERCC5*rs11069498 (b = −0.236, p = 0.044), *ERCC5*rs751402 (b = −0.224, p = 0.050), *ERCC5*rs873601 (b = −0.227, p = 0.037), and *SEPP1*rs3877899 (b = −0.327, p = 0.005). For concentration, possession of one or more minor alleles for every *SOD2* polymorphism evaluated, *SOD2*rs4880 (b = −0.303, p = 0.024), *SOD2*rs5746136 (b = −0.257, 0.023), *SOD2*rs8031 (b = −0.332, p = 0.011), contributed to poorer concentration performance regardless of group membership. In addition, the combination of Group B membership and possession of one or more *ERCC2*rs3916874 (b = 0.533, p = 0.050), *ERCC2*rs50872 (b = −0.882, p = 0.001), *ERCC3*rs4150407 (b = 0.546, p = 0.047), or *ERCC5*rs2296147 (b = 0.585, p = 0.043) minor alleles contributed positively or negatively to concentration scores. The combination of DNA repair gene variation and Group A membership was found to be associated with executive function performance with multiple significant SNP-by-Group A interaction effects observed: *ERCC3*rs2134794 (b = 0.470, p = 0.023), *ERCC3*rs4150407 (b = −0.466, p = 0.035), *ERCC3*rs4150477 (b = −0.417, p = 0.046), *ERCC5*rs2296147 (b = 0.477, p = 0.034), and *PARP1*rs2271347 (b = −0.589, p = 0.006). In contrast, the combination of Group B membership and possession of one or more *ERCC5*rs2296148 (b = 1.075, p = 0.024) or *SOD1*rs1041740 (b = −0.619, p = 0.015) minor alleles was associated with psychomotor speed scores. The combination of group membership and genetic variation was found to be important for all three memory-related cognitive composites as well. Specifically, the combination of Group A membership and possession of one or more minor alleles for: *ERCC5*rs11069498 contributed positively to verbal memory (b = 0.536, p = 0.034) and visual working memory (b = 0.629, p = 0.027) scores; *ERCC5*rs4150360 contributed positively to verbal memory (b = 0.568, p = 0.031) and visual working memory (b = 0.673, p = 0.023); and *ERCC5*rs751402 contributed positively to verbal memory (b = 0.486, p = 0.038) and visual memory (b = 0.499, p = 0.023). Additionally, the combination of Group A membership and *CAT* variation was found to be associated with visual memory: *CAT*rs1001179 (b = −0.512, p = 0.032), and *CAT*rs769214 (b = 0.480, p = 0.024). Two *CAT* SNP main effects, *CAT*rs525938 (b = −0.282, p = 0.049) and *CAT*rs566979 (b = −0.282, p = 0.049) were also observed with visual memory.

**Table 3:** Genetic risk score (GRS) and cognitive function composite regression analysis results

Composite cognitive function composite	Gene-SNP used in GRS calculation	Minor allele	Wildtype reference allele	b_GRS_	Model R^2^	R^2^ change for GRS
Attention^a^ (n = 214)	*ERCC3*-rs2134794	C	A	1.003*	0.236	0.048
	*ERCC5*-rs873601	G	A			
Concentration (n = 206)	*ERCC2*-rs3916874	C	G	0.619*	0.218	0.150
	*ERCC2*-rs50872	T	C			
	*ERCC3*-rs2134794	C	A			
	*ERCC3*-rs4150407	G	A			
	*ERCC5*-rs2296147	C	T			
	*SOD2*-rs4880	T	C			
	*SOD2*-rs5746136	A	G			
	*SOD2*-rs8031	T	A			
Executive function^a^ (n = 215)	*ERCC3*-rs2134794	C	A	0.535*	0.299	0.075
	*ERCC3*-rs4150407	G	A			
	*ERCC3*-rs4150477	T	C			
	*ERCC5*-rs2296147	C	T			
	*PARP1*-rs2271347	A	G			
	*PARP1*-rs3219058	A	G			
Mental flexibility^a^ (n = 198)	*ERCC2*-rs13181	G	T	0.669*	0.342	0.094
	*ERCC3*-rs4150407	G	A			
	*ERCC3*-rs4150477	T	C			
	*PARP1*-rs2271347	C	T			
	*SEPP1*-rs230819	A	C			
	*SEPP1*-rs3877899	A	G			
	*SOD1*-rs1041740	T	C			
Psychomotor speed^a^ (n = 186)	*CAT*-rs511895	A	G	0.741*	0.288	0.126
	*CAT*-rs769214	G	A			
	*ERCC5*-rs11069498	G	A			
	*ERCC5*-rs2296148	T	C			
	*ERCC5*-rs751402	T	C			
	*ERCC5*-rs873601	G	A			
	*SEPP1*-rs3877899	A	G			
	*SOD1*-rs1041740	T	C			
Verbal memory (n = 214)	*CAT*-rs566979	G	T	0.567*	0.289	0.049
	*ERCC5*-rs11069498	G	A			
	*ERCC5*-rs4150360	C	T			
	*ERCC5*-rs751402	T	C			
Visual memory^a^ (n = 178)	*CAT*-rs1001179	A	G	0.691*	0.260	0.118
	*CAT*-rs525938	G	A			
	*CAT*-rs566979	G	T			
	*CAT*-rs769214	G	A			
	*ERCC5*-rs751402	T	C			
	*GPX1*-rs1050450	G	A			
Visual working memory^a^ (n = 210)	*ERCC2*-rs1799787	T	C	0.766*	0.256	0.111
	*ERCC5*-rs11069498	G	A			
	*ERCC5*-rs4150355	T	C			
	*ERCC5*-rs4150360	C	T			
	*ERCC5*-rs873601	G	A			
	*PARP1*-rs2271347	C	T			

*SNP* single nucleotide polymorphism, *GRS* genetic risk score. All regression models are adjusted for age, estimated verbal intelligence, and levels of depression, anxiety, fatigue, and pain

* p < .001

^a^GRS calculation based on b-coefficients from regression models with modified influential point values

**Fig. 1:**
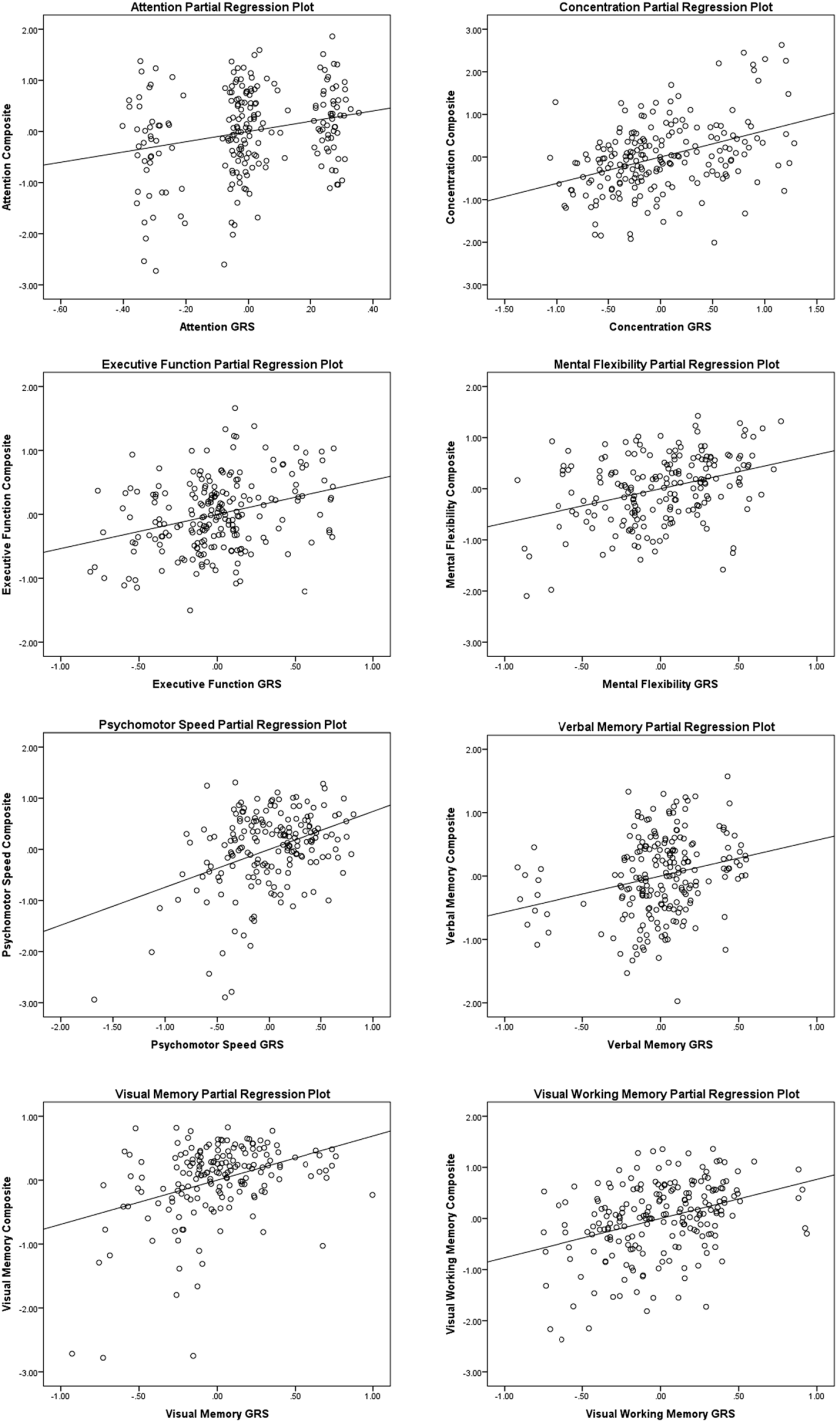
Genetic risk score (GRS) partial regression plots. *Note* The partial regression plots were generated using IBM^®^ SPSS^®^ Statistics Version 23 (IBM Corp., Armonk, NY). *GRS* genetic risk score

Each computed GRS was found to be significantly (p < 0.001) related to its respective CF composite (Table 3). All associations were found to be positive such that as GRSs increase, CF composite performance scores increase as well (Fig. 1).

## Discussion

To our knowledge, this study represents the first investigation of relationships between oxidative stress and DNA repair gene variation and pre-adjuvant therapy CF in postmenopausal women diagnosed with early-stage breast cancer. Overall, our results revealed that performance for every CF composite was significantly (p < 0.05) associated with one or more oxidative stress and DNA repair gene polymorphisms by either SNP main effects (i.e., observed cognitive changes in both breast cancer survivors and healthy control women are associated with a certain genetic polymorphism) and/or SNP-by-group interaction effects (i.e., observed cognitive changes were associated with a certain combination of genetic polymorphism and prescribed treatment group).

Out of all the genes included in our investigation, variation in *ERCC5* appeared to influence cognitive performance most globally, with significant relationships noted between one or more *ERCC5* SNPs and every CF composite with the exception of mental flexibility. The function of *ERCC5* has been most widely investigated in xeroderma pigmentosum and DNA excision repair following UV-induced damage. More generally, *ERCC5* participates in nucleotide excision repair, encoding an endonuclease that makes 3’ incisions (US National Library of Medicine, National Institutes of Health 2014). *ERCC5* also decreases cellular oxidative burden, functioning as a cofactor for a DNA glycosylase that removes oxidized pyrimidines from DNA (US National Library of Medicine, National Institutes of Health 2014). Although rare, mutations in the *ERCC5* gene have also been associated with the development of Cockayne syndrome in combination with xeroderma pigmentosum (US National Library of Medicine, National Institutes of Health 2014). Characteristic features of Cockayne syndrome include impaired nervous system development and mental retardation, highlighting the critical role *ERCC5* plays in normal CF. Within the context of this study, we postulate that as cancer creates a cellular environment of increased oxidative stress and DNA damage, certain polymorphisms in *ERCC5* may decrease a survivor’s ability to repair damage and remove reactive oxygen species, placing an already vulnerable brain at even higher risk for damage (Conroy et al. 2012; Joshi et al. 2005; Kasapović et al. 2010; Walker et al. 2012; Finkel and Holbrook 2000).

Another intriguing finding was the predominant negative effect of certain *SOD2* alleles (rs4880-T, rs5746136-A, and rs8031-T) on concentration performance within our study sample regardless of cancer diagnosis or prescribed treatment group. The *SOD2* gene encodes an enzyme, manganese-dependent superoxide dismutase, that confers cell protection by eliminating mitochondrial reactive oxygen species (NCBI Resource Coordinators 2015). The functional rs4880 alanine-to-valine (C > T) single amino acid change has been found to influence enzyme activity with valine (T) associated with reduced *SOD2* activity in human breast cancer and hepatoma cell lines (Sutton et al. 2005; McAteea and Yager 2010); contradictory findings have also been reported (Martin et al. 2009; Bastaki et al. 2006). Nevertheless, reduced *SOD2* expression has been implicated in a number of neurodegenerative disorders (Flynn and Melov 2013). Likewise, decreased *SOD2* mRNA and protein levels were found to be correlated with poorer memory, attention span, verbal fluency, and learning ability in a pooled sample of adults with recurrent depressive disorder and healthy controls (Talarowska et al. 2014). As our study was not designed to measure expression or protein levels of *SOD2*, we cannot expand upon how our results support or refute previous findings. While we found that possession of one or more *SOD2* rs4880-T alleles was associated with poorer CF in all study participants, the antioxidant properties of *SOD2* may have more impactful consequences for women with breast cancer throughout treatment with the introduction of adjuvant therapy regimens known to increase oxidative burden systemically. Thus, a remaining important question is if possession of one or more SOD2 rs4880-T alleles is also associated with greater risk for cognitive decline with therapy.

Our analyses also revealed a number of significant allele effects specific to the groups of women with breast cancer compared to control women without cancer. For example, significant SNP main effects and SNP-by-group interaction effects were observed for *PARP1* rs2271347 and executive function performance. While the SNP effect regression coefficient for possession of one or more minor alleles contributed positively to executive function (b = 0.502, p < 0.001), the SNP-by-group interaction regression coefficients for women in Group A (scheduled to received chemotherapy plus anastrozole) (b = −0.589, p = 0.006) and women in Group B (scheduled to receive anastrozole alone) (b = −0.498, p = 0.006) contributed negatively to the model, nullifying the main effect and contributing an overall negative input to executive function performance. Interactions, like the one presented, illustrate how not only genetic variation, but the combination of genetic variation and a breast cancer diagnosis can impact CF at diagnosis; alternatively, these findings highlight how factors that increase one’s risk for development of cancer may also contribute to changes in CF.

In order to get a sense of the effect of the oxidative stress and DNA repair candidate pathway as a whole on pretreatment CF in women with breast cancer, we calculated weighted GRSs for each CF composite based on our individual SNP analysis. Similar to how a total score from an instrument (composed of individual items) summarizes a given concept, GRSs “summarize the potential multiple risk genetic influences into a single quantitative parameter and do not depend on single genetic variants” (Carreras-Torres et al. 2014). Instead of using a simple count method where each SNP contributes equally to risk calculations, we employed a weighted method in order to assign greater risk/protection to minor alleles with stronger associations (Lu et al. 2010). Fascinatingly, each computed GRS was significantly and positively associated to its respective CF composite. The amount of explained variance that each GRS contributed to its respective model was also notable, ranging from R^2^ = 0.048 to 0.150. These findings not only point to the potential importance of oxidative stress response and DNA repair capacity to pretreatment changes in CF in breast cancer survivors, but, more broadly, to the value of evaluating the effect of multiple SNPs at the same time in association studies in general.

To better interpret our findings, a gene–gene pathway analysis, using Ingenuity^®^ Pathway Analysis software (IPA^®^, QIAGEN Redwood City, www.qiagen.com/ingenuity), of the nine candidate oxidative stress and DNA repair genes analyzed within this study was conducted. This analysis reiterated the interconnectedness of the genes and, consequently, endorsed evaluation of the collective effect of multiple SNPs from a single pathway simultaneously. Two unique networks were identified through our analysis (Additional file 2). The first network included *CAT*, *GPX1*, *PARP1*, *SOD1*, and *SOD2*. The main associated diseases and functions of this network were, not surprisingly, free radical scavenging, small molecule biochemistry, and neurological disease. The second network included *ERCC2*, *ERCC3*, *ERCC5*, and *SEPP1* and was associated with DNA replication, recombination, and repair, energy production, and nucleic acid metabolism. The pathway analysis also reminds that our study featured a limited number of candidate genes and that there are many additional oxidative stress and DNA repair genes that warrant further investigation.

While this study had a number of strengths, including mechanistic pathway-driven candidate gene selection, inclusion of a matched control group of women without a breast cancer diagnosis, assessment of SNP-by-prescribed treatment group interaction effects, and evaluation of the collective effect of multiple SNPs using weighted GRSs, limitations should also be acknowledged. To begin, the small study sample size, while appreciable, did not allow for detection of small effect sizes (i.e., R^2^ < .01) often characteristic of genetic association studies. In addition, the small sample size did not allow for the evaluation of allelic dose–response relationships. Because the sample was comprised of postmenopausal women with hormone receptor positive, early-stage breast cancer who were primarily white and married, the generalizability to other more diverse populations and breast cancers is unknown. Findings from this study should be replicated in a larger, more diverse sample. In addition, differences in anesthesia exposure (or lack thereof for control women who did not undergo surgery) and its potential confounding cognitive effects were not controlled for in our analysis. Future studies and analyses should also focus on the collective effect of multiple oxidative stress and DNA repair gene variants on CF throughout and following completion of adjuvant therapy in women with breast cancer.

In conclusion, our goal in this study was to provide data on a possible biologic mechanism to account for variability in cognitive changes in breast cancer survivors. Results from this preliminary study reveal that genetic variation in the oxidative stress and DNA repair pathways appears to play an important role in CF in women with breast cancer prior to initiation of adjuvant therapy and give reason to investigate whether polymorphisms influence cognitive decline with therapy as well. In the future, evaluation of a panel of oxidative stress and DNA repair gene polymorphisms could offer healthcare providers a means of predicting which women diagnosed breast cancer are most at risk for poorer CF and candidates for additional interventions, such as antioxidant therapy.

## Additional files

10.1186/s40064-016-2061-4 Individual SNP and cognitive function regression analyses.
10.1186/s40064-016-2061-4 Oxidative stress and DNA repair gene-gene networks generated by pathway analysis.

## Abbreviations

BDI-II: Beck Depression Inventory-II
BPI: Brief Pain Inventory
CAT: catalase
CF: cognitive function
ERCC2: Excision Repair Cross-Complementation Group 2
ERCC3: Excision Repair Cross-Complementation Group 3
ERCC5: Excision Repair Cross-Complementation Group 5
GPX1: Glutathione Peroxidase 1
GRS: genetic risk score
NART-R: National Adult Reading Test-Revised
PARP1: Poly (ADP-ribose) Polymerase 1
PBL: peripheral blood leukocytes
POMS: Profile of Mood States
SD: standard deviation
SEPP1: Selenoprotein P, Plasma 1
SNP: single nucleotide polymorphism
SOD1: Superoxide Dismutase 1, Soluble
SOD2: Superoxide Dismutase 2, Mitochondrial

## References

[CR1] Ahles TA, Saykin AJ (2007). Candidate mechanisms for chemotherapy-induced cognitive changes. Nat Rev Cancer.

[CR2] Ahles TA, Root JC, Ryan EL (2012). Cancer- and cancer treatment-associated cognitive change: an update on the state of the science. J Clin Oncol.

[CR3] Bastaki M, Huen K, Manzanillo P, Chande N, Chen C, Balmes JR, Tager IB, Holland N (2006). Genotype–activity relationship for Mn-superoxide dismutase, glutathione peroxidase 1 and catalase in humans. Pharmacogenet Genomics.

[CR4] Beck AT, Steer RA, Brown GK (1996). Beck Depression Inventory-II.

[CR5] Bender CM, Merriman JD, Gentry AL, Ahrendt GM, Berga SL, Brufsky AM, Casillo FE, Dailey MM, Erickson KI, Kratofil FM, McAuliffe PF, Rosenzweig MQ, Ryan CM, Sereika SM (2015). Patterns of change in cognitive function with anastrozole therapy. Cancer.

[CR6] Blasiak J, Arabski M, Krupa R, Wozniak K, Rykala J, Kolacinska A, Morawiec Z, Drzewoski J, Zadrozny M (2004). Basal, oxidative and alkylative DNA damage, DNA repair efficacy and mutagen sensitivity in breast cancer. Mutat Res.

[CR7] Bucholtz N, Demuth I (2013). DNA-repair in mild cognitive impairment and Alzheimer’s disease. DNA Repair (Amst).

[CR8] Carreras-Torres R, Kundu S, Zanetti D, Esteban E, Via M, Moral P (2014). Genetic Risk score of NOS gene variants associated with myocardial infarction correlates with coronary incidence across Europe. PLoS ONE.

[CR9] Cleeland CS, Chapman CR, Loeser JD (1989). Measurement of pain by subjective report. Advances in pain research and therapy.

[CR10] Cockburn J, Smith PT (1993). Correlates of everyday memory among residents of Part III homes. Br J Clin Psychol.

[CR11] Conroy SK, McDonald BC, Smith DJ, Moser LR, West JD, Kamendulis LM, Klaunig JE, Champion VL, Unverzagt FW, Saykin AJ (2012). Alterations in brain structure and function in breast cancer survivors: effect of post-chemotherapy interval and relation to oxidative DNA damage. Breast Cancer Res Treat.

[CR12] Coppedè F, Migliore L (2010). DNA repair in premature aging disorders and neurodegeneration. Curr Aging Sci.

[CR13] De Haan JB, Griffiths P, Kelner M, Shea RDO, Cheung NS, Bronson RT, Silvestro MJ, Wild S, Zheng SS, Beart PM, Hertzog PJ, Kola I (1998). Mice with a homozygous null mutation for the most abundant glutathione peroxidase, Gpx1, show increased Susceptibility to the oxidative stress-inducing agents paraquat and hydrogen peroxide. J Biol Chem.

[CR14] Delis DC, Kaplan E, Kramer JH (2001). Delis-Kaplan (D-KEFS) executive function system, examiners manual.

[CR15] DNA Genotek Inc (2012). Laboratory protocol for manual purification of DNA from whole sample.

[CR16] Edge SB, Byrd DR, Compton CC, Fritz AG, Greene FL, Trotti A (2010). AJCC Cancer Staging Manual.

[CR17] Finkel T, Holbrook NJ (2000). Oxidants, oxidative stress and the biology of ageing. Nature.

[CR18] Flynn JM, Melov S (2013). SOD2 in mitochondrial dysfunction and neurodegeneration. Free Radic Biol Med.

[CR19] Hamanishi T, Furuta H, Kato H, Doi A, Tamai M, Shimomura H, Sakagashira S, Nishi M, Sasaki H, Sanke T, Nanjo K (2004). Functional variants in the glutathione peroxidase-1 (GPx-1) gene are associated with increased intima-media thickness of carotid arteries and risk of macrovascular diseases in Japanese type 2 diabetic patients. Diabetes.

[CR20] Herrera ACS, Victorino VJ, Campos FC, Verenitach BD, Lemos LT, Aranome AMF, Oliveira SR, Cecchini AL, Simão ANC, Abdelhay E, Panis C, Cecchini R (2014). Impact of tumor removal on the systemic oxidative profile of patients with breast cancer discloses lipid peroxidation at diagnosis as a putative marker of disease recurrence. Clin Breast Cancer.

[CR21] Hooker S, Bonilla C, Akereyeni F, Ahaghotu C, Kittles R (2007). NAT2 and NER genetic variants and sporadic prostate cancer susceptibility in African Americans. Prostate Cancer Prostatic Dis..

[CR22] Islam T, Mcconnell R, Gauderman WJ, Avol E, Peters JM, Gilliland FD (2007). Ozone, oxidant defense genes, and risk of asthma during adolescence. Am J Respir Crit Care Med.

[CR23] Janelsins MC, Kohil S, Mohile SG, Usuki K, Ahles T, Morrow GR (2012). An update on cancer- and chemotherapy-related cognitive dysfunction. Semin Oncol.

[CR24] Jeppesen D, Bohr V, Stevnsner T (2012). DNA repair deficiency in neurodegeration. Prog Neurobiol.

[CR25] Jezierska-Drutel A, Rosenzweig S, Neumann C (2013). Role of oxidative stress and the microenvironment in breast cancer development and progression. Adv Cancer Res.

[CR26] Jiang Z, Akey JM, Shi J, Xiong M, Wang Y, Shen Y, Xu X, Chen H, Wu H, Xiao J, Lu D, Huang W, Jin L (2001). A polymorphism in the promoter region of catalase is associated with blood pressure levels. Hum Genet.

[CR27] Jones S, Nee L, Sweet L, Polinsky R, Bartlett J, Bradley W, Robinson S (1989). Decreased DNA repair in familial Alzheimer’s disease. Mutat Res.

[CR28] Joshi G, Sultana R, Tangpong J, Cole MP, St Clair DK, Vore M, Estus S, Butterfield DA (2005). Free radical mediated oxidative stress and toxic side effects in brain induced by the anti cancer drug adriamycin: insight into chemobrain. Free Radic Res.

[CR29] Kang D (2002). Oxidative stress, DNA damage, and breast cancer. AACN Clin Issues.

[CR30] Kasapović J, Pejić S, Stojiljković V, Todorović A, Radošević-Jelić L, Saičić ZS, Pajović SB (2010). Antioxidant status and lipid peroxidation in the blood of breast cancer patients of different ages after chemotherapy with 5-fluorouracil, doxorubicin and cyclophosphamide. Clin Biochem.

[CR31] Klove H (1963). Clinical neuropsychology.

[CR32] Layfayette Clinical Insturments Company (1989) Layfayette Clinical Repeatable Neuropsychological Battery

[CR33] Lillenes MS, Espeseth T, Støen M, Lundervold AJ, Frye SA, Rootwelt H, Reinvang I, Tønjum T (2011). DNA base excision repair gene polymorphisms modulate human cognitive performance and decline during normal life span. Mech Ageing Dev.

[CR34] Lockett KL, Hall MC, Xu J, Zheng SL, Berwick M, Chuang S, Clark PE, Cramer SD, Lohman K, Hu JJ (2004). The ADPRT V762A genetic variant contributes to prostate cancer susceptibility and deficient enzyme function. Cancer Res.

[CR35] Lu Y, Feskens EJM, Boer JMA, Imholz S, Verschuren WMM, Wijmenga C, Vaarhorst A, Slagboom E, Müller M, Dollé MET (2010). Exploring genetic determinants of plasma total cholesterol levels and their predictive value in a longitudinal study. Atherosclerosis.

[CR36] Martin RCG, Li Y, Liu Q, Jensen NS, Barker DF, Doll MA, Hein DW (2009). Manganese superoxide dismutase V16A single-nucleotide polymorphism in the mitochondrial targeting sequence is associated with reduced enzymatic activity in cryopreserved human hepatocytes. DNA Cell Biol.

[CR37] McAteea BL, Yager JD (2010). Manganese superoxide dismutase: effect of the ala16 val polymorphism on protein, activity, and mRNA levels in human breast cancer cell lines and stably transfected mouse embryonic fibroblasts. Mol Cell Biochem.

[CR38] McNair D, Lorr M, Droppleman LF (1992). EdITS manual for the profile of mood states.

[CR39] Meplan C, Crosley LK, Nicol F, Beckett GJ, Howie AF, Hill KE, Horgan G, Mathers JC, Arthur JR, Hesketh JE (2007). Genetic polymorphisms in the human selenoprotein P gene determine the response of selenoprotein markers to selenium supplementation in a gender-specific manner (the SELGEN study). FASEB J.

[CR40] Migliore L, Fontana I, Trippi F, Colognato R, Coppedè F, Tognoni G, Nucciarone B, Siciliano G (2005). Oxidative DNA damage in peripheral leukocytes of mild cognitive impairment and AD patients. Neurobiol Aging.

[CR41] Miller SA, Dykes DD, Polesky HF (1988). A simple salting out procedure for extracting DNA from human nucleated cells. Nucleic Acids Res.

[CR42] Mizutani H (2007). Mechanism of DNA damage and apoptosis induced by anticancer drugs through generation of reactive oxygen species. Yakugaku Zasshi.

[CR43] Nadin SB, Vargas-Roig LM, Drago G, Ibarra J, Ciocca DR (2006). DNA damage and repair in peripheral blood lymphocytes from healthy individuals and cancer patients: a pilot study on the implications in the clinical response to chemotherapy. Cancer Lett.

[CR44] NCBI Resource Coordinators (2015). Database resources of the National Center for Biotechnology Information. Nucleic Acids Res.

[CR45] Nelson H (1981). Nelson Adult Reading Test (NART) manual.

[CR46] Nourazarian A, Kangari P, Salmaninejad A (2014). Roles of oxidative stress in the development and progression of breast cancer. Asian Pac J Cancer Prev.

[CR47] Osterrieth PA (1944). Test of copying a complex figure: contribution to the study of perception and memory. Arch Psychol (Geneve).

[CR48] Panis C, Victorino VJ, Herrera ACS, Freitas LF, De Rossi T, Campos FC, Simão AN, Barbosa DS, Pinge-Filho P, Cecchini R, Cecchini AL (2012). Differential oxidative status and immune characterization of the early and advanced stages of human breast cancer. Breast Cancer Res Treat.

[CR49] Rey A (1964). L’examen psychologique dans les cas d’encephalopathie traumatique. Arch Psychol.

[CR50] Robbins T, James M, Owen A, Sahakian B, McInnes L, Rabbitt P (1994). Cambridge neuropsychological test automated battery (CANTAB): a factor analytic study of a large sample of normal elderly volunteers. Dementia.

[CR51] Saintot M, Mathieu-Daude H, Astre C, Grenier J, Simony-Lafontaine J, Gerber M (2002). Oxidant–antioxidant status in relation to survival among breast cancer patients. Int J Cancer.

[CR52] Sánchez-Suárez P, Ostrosky-Wegman P, Gallegos-Hernández F, Peñarroja-Flores R, Toledo-García J, Bravo JL, Del Castillo ER, Benítez-Bribiesca L (2008). DNA damage in peripheral blood lymphocytes in patients during combined chemotherapy for breast cancer. Mutat Res.

[CR53] Spencer DMS, Bilardi RA, Koch TH, Post GC, Nafie JW, Kimura KI, Cutts SM, Phillips DR (2008). DNA repair in response to anthracycline-DNA adducts: a role for both homologous recombination and nucleotide excision repair. Mutat Res.

[CR54] Sutton A, Imbert A, Igoudjil A, Descatoire V, Cazanave S, Pessayre D, Degoul F (2005). The manganese superoxide dismutase Ala16Val dimorphism modulates both mitochondrial import and mRNA stability. Pharmacogenet Genomics.

[CR55] Talarowska M, Orzechowska A, Szemraj J, Su KP, Maes M, Galecki P (2014). Manganese superoxide dismutase gene expression and cognitive functions in recurrent depressive disorder. Neuropsychobiology.

[CR56] US National Library of Medicine, National Institutes of Health (2014) Genetics Home Reference: ERCC5. http://ghr.nlm.nih.gov/gene/ERCC5. Accessed 26 Jan 2016

[CR57] Valenti L, Conte D, Piperno A, Dongiovanni P, Fracanzani AL, Fraquelli M, Vergani A, Gianni C, Carmagnola L, Fargion S (2004). The mitochondrial superoxide dismutase A16 V polymorphism in the cardiomyopathy associated with hereditary haemochromatosis. J Med Genet.

[CR58] Vardy J, Wefel JS, Ahles T, Tannock IF, Schagen SB (2008). Cancer and cancer-therapy related cognitive dysfunction: an international perspective from the Venice cognitive workshop. Ann Oncol.

[CR59] Walker CH, Drew BA, Antoon JW, Kalueff AV, Beckman BS (2012). Neurocognitive effects of chemotherapy and endocrine therapies in the treatment of breast cancer: recent perspectives. Cancer Invest.

[CR60] Wang C, Yu J, Wang H, Zhang J, Wu N (2014). Lipid peroxidation and altered antioxidant status in breast adenocarcinoma patients. Drug Res (Stuttg).

[CR61] Wechsler D (1998). The Wechsler memory scale-revised.

[CR62] Wefel JS, Witgert ME, Meyers CA (2008). Neuropsychological sequelae of non-central nervous system cancer and cancer therapy. Neuropsychology.

