# Supplementary material for: Polymorphisms in DNA repair and oxidative stress genes associated with pre-treatment cognitive function in breast cancer survivors: an exploratory study
Source: Springerplus. 2016 Apr 9;5:422. doi: 10.1186/s40064-016-2061-4 (PMC4826652; doi:10.1186/s40064-016-2061-4)
Supplement: Supplementary file 1 — 10.1186/s40064-016-2061-4 Individual SNP and cognitive function regression analyses. [file 40064_2016_2061_MOESM1_ESM.pdf]

Polymorphisms in DNA repair and oxidative stress genes associated with pre-treatment cognitive function in breast cancer survivors: an exploratory study

Breast Cancer Research and Treatment

Theresa A. Koleck[1] (tat30@pitt.edu), Catherine M. Bender[1], Susan M. Sereika[1,2], Adam M. Brufsky[3-5], Barry C. Lembersky[3,4], Priscilla F. McAuliffe[5,6], Shannon L. Puhalla[3,5], Priya Rastogi[3,5] & Yvette P. Conley[1,7]

[1]School of Nursing, University of Pittsburgh, 3500 Victoria Street, Pittsburgh, PA 15261, USA
[2]Department of Biostatistics and Department of Epidemiology, Graduate School of Public Health, University of Pittsburgh, 130 De Soto Street, Pittsburgh, PA 15261, USA
[3]Division of Hematology/Oncology, Magee-Womens Hospital of University of Pittsburgh Medical Center (UPMC), 300 Halket Street, Pittsburgh, PA 15213, USA
[4]University of Pittsburgh Cancer Institute, 5150 Centre Avenue, Pittsburgh, PA 15232, USA
[5]School of Medicine, University of Pittsburgh, 3550 Terrace Street, Pittsburgh, PA 15261, USA
[6]Magee-Womens Surgical Associates, Magee-Womens Hospital of University of Pittsburgh Medical Center (UPMC), 300 Halket Street, Pittsburgh, PA 15213, USA
[7]Department of Human Genetics, Graduate School of Public Health, University of Pittsburgh, 130 De Soto Street, Pittsburgh, PA 15261, USA

Online Resource 1 Individual SNP and cognitive function regression analysis

b-coefficient, p-value	Attention[a]	Concentration	Executive Function[a]	Mental Flexibility[a]	Psychomotor Speed[a]	Verbal Memory	Visual Memory[a]	Visual Working Memory[a]
CATrs1001179, AA+GA vs. GG								
Main Effects Model	n=187	n=187	n=188	n=187	n=188	n=188	n=188	n=188
SNP Main Effect	0.025, 0.856	-0.066, 0.614	0.078, 0.381	-0.014, 0.893	0.153, 0.201	-0.043, 0.668	0.027, 0.777	-0.062, 0.585
Interaction Model								
SNP Main Effect	0.159, 0.478	-0.096, 0.651	0.199, 0.167	-0.088 , 0.611	0.228, 0.243	-0.074, 0.65	0.234, 0.124	-0.211, 0.249
SNP-by-GroupA Inter	-0.145, 0.683	0.222, 0.509	-0.046, 0.837	-0.075, 0.781	-0.137, 0.652	0.081, 0.749	-0.512, 0.032*	0.117, 0.683
SNP-by-GroupB Inter	-0.253, 0.42	-0.052, 0.861	-0.278, 0.168	0.231, 0.342	-0.107, 0.695	0.03, 0.894	-0.222, 0.299	0.308, 0.229
CATrs10488736, TT+CT vs. CC								
Main Effects Model	n=217	n=217	n=218	n=217	n=218	n=218	n=218	n=218
SNP Main Effect	-0.01, 0.934	0.086, 0.453	-0.038, 0.632	0.106, 0.241	0.093, 0.396	-0.051, 0.559	0.012, 0.888	-0.061, 0.54
Interaction Model								
SNP Main Effect	0.024, 0.904	0.001, 0.996	-0.006, 0.967	0.107, 0.484	0.286, 0.119	-0.162, 0.271	0.054, 0.699	-0.05, 0.763
SNP-by-GroupA Inter	-0.251, 0.416	-0.091, 0.76	0.023, 0.91	0.002, 0.994	-0.423, 0.135	0.089, 0.696	-0.145, 0.5	0.129, 0.616
SNP-by-GroupB Inter	0.075, 0.787	0.278, 0.3	-0.1, 0.595	-0.003, 0.99	-0.222, 0.385	0.229, 0.268	-0.014, 0.944	-0.112, 0.63
CATrs2179625, GG+TG vs. TT								
Main Effects Model	n=217	n=217	n=218	n=217	n=218	n=218	n=218	n=218

SNP Main Effect	0.146, 0.219	0.105, 0.358	-0.073, 0.358	0.162, 0.077	0.075, 0.494	-0.09, 0.306	0.078, 0.346	-0.11, 0.265
Interaction Model								
SNP Main Effect	0.199, 0.307	0.04, 0.831	-0.152, 0.244	0.234, 0.122	0.269, 0.135	-0.221, 0.127	0.127, 0.352	-0.177, 0.278
SNP-by-GroupA Inter	-0.431, 0.16	-0.058, 0.845	0.243, 0.235	-0.033, 0.887	-0.42, 0.136	0.229, 0.311	-0.198, 0.352	0.317, 0.215
SNP-by-GroupB Inter	0.143, 0.6	0.208, 0.43	0.048, 0.794	-0.167, 0.429	-0.235, 0.353	0.193, 0.34	0.003, 0.989	-0.035, 0.878
CATrs511895, AA+GA vs. GG								
Main Effects Model	n=214	n=214	n=215	n=214	n=215	n=215	n=215	n=215
SNP Main Effect	0.196, 0.105	0.071, 0.54	0.083, 0.298	0.019, 0.84	0.237, 0.031*	0.146, 0.1	0.136, 0.106	0.067, 0.505
Interaction Model								
SNP Main Effect	0.273, 0.167	0.005, 0.977	0.161, 0.214	-0.096, 0.533	0.056, 0.754	0.241, 0.098	0.245, 0.072	0.044, 0.788
SNP-by-GroupA Inter	-0.082, 0.792	0.222, 0.454	-0.005, 0.978	0.206, 0.39	0.351, 0.21	-0.088, 0.696	-0.377, 0.077	0.151, 0.56
SNP-by-GroupB Inter	-0.149, 0.593	0.028, 0.917	-0.203, 0.27	0.165, 0.446	0.246, 0.33	-0.19, 0.352	-0.04, 0.834	-0.038, 0.87
CATrs525938, GG+AG vs. AA								
Main Effects Model	n=216	n=216	n=217	n=216	n=217	n=217	n=217	n=217
SNP Main Effect	-0.198, 0.093	-0.042, 0.711	0.007, 0.925	-0.029, 0.751	-0.189, 0.081	-0.032, 0.719	-0.138, 0.093	-0.02, 0.839
Interaction Model								
SNP Main Effect	-0.355, 0.067	0.129, 0.489	-0.075, 0.562	0.086, 0.567	-0.279, 0.118	0.019, 0.895	-0.332, 0.014*	-0.025, 0.875
SNP-by-GroupA Inter	0.18, 0.56	-0.209, 0.483	0.047, 0.82	-0.28, 0.239	-0.036, 0.9	-0.207, 0.369	0.412, 0.053	-0.267, 0.299
SNP-by-GroupB Inter	0.296, 0.277	-0.314, 0.233	0.187, 0.307	-0.117, 0.579	0.259, 0.302	0.002, 0.993	0.241, 0.2	0.189, 0.407
CATrs566979, GG+TG vs. TT								
Main Effects Model	n=218	n=218	n=219	n=218	n=219	n=219	n=219	n=219
SNP Main Effect	-0.062, 0.613	0.073, 0.535	-0.077, 0.343	0.02, 0.834	-0.15, 0.179	-0.121, 0.181	-0.124, 0.141	-0.155, 0.126
Interaction Model								
SNP Main Effect	0.067, 0.745	0.122, 0.533	-0.113, 0.414	0.078, 0.621	-0.024, 0.896	-0.346, 0.023*	-0.282, 0.049*	-0.208, 0.224
SNP-by-GroupA Inter	-0.501, 0.12	-0.41, 0.187	0.1, 0.644	-0.135, 0.586	-0.508, 0.083	0.282, 0.234	0.147, 0.507	0.181, 0.498
SNP-by-GroupB Inter	-0.015, 0.958	0.12, 0.656	0.027, 0.885	-0.064, 0.77	-0.002, 0.993	0.387, 0.064	0.301, 0.124	0.021, 0.928
CATrs769214, GG+AG vs. AA								
Main Effects Model	n=214	n=214	n=215	n=214	n=215	n=215	n=215	n=215

SNP Main Effect	-0.151, 0.201	0.01, 0.928	-0.031, 0.695	-0.015, 0.872	-0.209, 0.057	0.006, 0.949	-0.156, 0.058	-0.025, 0.803
Interaction Model								
SNP Main Effect	-0.334, 0.088	0.23, 0.224	-0.162, 0.218	0.104, 0.494	-0.421, 0.02*	0.01, 0.943	-0.388, 0.004*	-0.06, 0.717
SNP-by-GroupA Inter	0.223, 0.47	-0.221, 0.46	0.118, 0.57	-0.191, 0.423	0.194, 0.494	-0.099, 0.667	0.48, 0.024*	-0.106, 0.682
SNP-by-GroupB Inter	0.333, 0.224	-0.433, 0.105	0.268, 0.149	-0.185, 0.386	0.429, 0.091	0.053, 0.797	0.29, 0.126	0.163, 0.484
ERCC2rs13181, GG+TG vs. TT								
Main Effects Model	n=218	n=218	n=219	n=218	n=219	n=219	n=219	n=219
SNP Main Effect	-0.03, 0.803	-0.039, 0.733	0.02, 0.8	-0.197, 0.031*	0.051, 0.642	0.019, 0.834	0.06, 0.468	0.032, 0.748
Interaction Model								
SNP Main Effect	0.04, 0.841	0.008, 0.967	0.05, 0.707	-0.204, 0.178	0.086, 0.636	-0.088, 0.548	0.144, 0.298	0.067, 0.683
SNP-by-GroupA Inter	0.11, 0.722	0.058, 0.844	0.091, 0.658	0.06, 0.796	-0.094, 0.739	0.241, 0.293	-0.141, 0.511	0.061, 0.812
SNP-by-GroupB Inter	-0.254, 0.358	-0.16, 0.545	-0.138, 0.454	-0.022, 0.917	-0.029, 0.908	0.118, 0.564	-0.124, 0.518	-0.134, 0.561
ERCC2rs1799786, TT+CT vs. CC								
Main Effects Model	n=188	n=188	n=189	n=189	n=189	n=189	n=189	n=189
SNP Main Effect	0.15, 0.25	-0.078, 0.515	0.086, 0.302	-0.136, 0.166	-0.024, 0.84	0.074, 0.434	0.019, 0.819	0.073, 0.501
Interaction Model								
SNP Main Effect	0.155, 0.475	-0.006, 0.975	0.118, 0.398	-0.01, 0.951	0.087, 0.66	0.025, 0.874	0.056, 0.693	0.315, 0.083
SNP-by-GroupA Inter	-0.143, 0.674	0.12, 0.698	0.053, 0.806	-0.211, 0.407	-0.154, 0.616	0.102, 0.68	-0.133, 0.548	-0.384, 0.174
SNP-by-GroupB Inter	0.081, 0.79	-0.267, 0.339	-0.119, 0.545	-0.189, 0.413	-0.188, 0.502	0.06, 0.788	-0.007, 0.974	-0.377, 0.142
ERCC2rs1799787, TT+CT vs. CC								
Main Effects Model	n=218	n=218	n=219	n=218	n=219	n=219	n=219	n=219
SNP Main Effect	0.06, 0.611	-0.131, 0.244	0.104, 0.186	-0.135, 0.133	0.072, 0.504	0.101, 0.246	0.092, 0.26	0.139, 0.155
Interaction Model								
SNP Main Effect	0.131, 0.5	-0.013, 0.944	0.231, 0.074	-0.066, 0.658	0.139, 0.435	0.064, 0.656	0.248, 0.065	0.328, 0.041*
SNP-by-GroupA Inter	-0.004, 0.99	0.081, 0.781	-0.125, 0.539	-0.113, 0.628	-0.135, 0.63	0.109, 0.63	-0.263, 0.213	-0.203, 0.42
SNP-by-GroupB Inter	-0.184, 0.5	-0.362, 0.161	-0.253, 0.163	-0.107, 0.607	-0.087, 0.729	0.025, 0.9	-0.236, 0.21	-0.364, 0.107
ERCC2rs238406, TT+GT vs. GG								
Main Effects Model	n=213	n=213	n=214	n=213	n=214	n=214	n=214	n=214

SNP Main Effect	-0.023, 0.856	0.097, 0.415	0.034, 0.681	-0.015, 0.881	-0.104, 0.363	0.041, 0.654	0.021, 0.802	0.019, 0.859
Interaction Model								
SNP Main Effect	-0.027, 0.895	0.075, 0.691	0.055, 0.683	-0.121, 0.438	-0.139, 0.443	0.15, 0.311	0.13, 0.32	-0.242, 0.147
SNP-by-GroupA Inter	-0.045, 0.892	-0.014, 0.964	0.036, 0.868	0.106, 0.674	0.101, 0.732	-0.158, 0.51	-0.081, 0.702	0.444, 0.102
SNP-by-GroupB Inter	0.039, 0.893	0.07, 0.799	-0.08, 0.676	0.221, 0.322	0.031, 0.907	-0.193, 0.365	-0.249, 0.187	0.421, 0.08
ERCC2rs238416, AA+GA vs. GG								
Main Effects Model	n=216	n=216	n=217	n=216	n=217	n=217	n=217	n=217
SNP Main Effect	-0.051, 0.671	0.125, 0.281	-0.066, 0.414	0.049, 0.594	0.034, 0.76	-0.048, 0.595	0.058, 0.492	0.004, 0.965
Interaction Model								
SNP Main Effect	0.071, 0.712	0.163, 0.379	-0.048, 0.711	-0.032, 0.832	0.053, 0.766	-0.137, 0.342	0.16, 0.235	-0.05, 0.756
SNP-by-GroupA Inter	-0.352, 0.256	-0.252, 0.395	-0.011, 0.956	0.099, 0.676	0.021, 0.941	0.304, 0.185	-0.023, 0.913	0.423, 0.098
SNP-by-GroupB Inter	-0.095, 0.734	0.071, 0.793	-0.042, 0.824	0.156, 0.471	-0.07, 0.788	0.032, 0.877	-0.271, 0.165	-0.152, 0.514
ERCC2rs3916874, CC+GC vs. GG								
Main Effects Model	n=211	n=211	n=212	n=212	n=212	n=212	n=212	n=212
SNP Main Effect	-0.088, 0.467	-0.012, 0.916	-0.016, 0.838	-0.049, 0.601	0.002, 0.989	-0.033, 0.716	-0.07, 0.41	-0.06, 0.559
Interaction Model								
SNP Main Effect	-0.203, 0.314	-0.288, 0.139	0.117, 0.384	-0.084, 0.59	-0.017, 0.928	0.021, 0.889	-0.005, 0.975	0.064, 0.705
SNP-by-GroupA Inter	0.429, 0.166	0.278, 0.352	-0.304, 0.141	0.068, 0.775	0.131, 0.651	-0.19, 0.413	-0.098, 0.654	-0.24, 0.358
SNP-by-GroupB Inter	0.008, 0.979	0.533, 0.05*	-0.142, 0.451	0.046, 0.832	-0.042, 0.873	-0.011, 0.96	-0.105, 0.598	-0.16, 0.501
ERCC2rs50871, GG+TG vs. TT								
Main Effects Model	n=217	n=217	n=218	n=217	n=218	n=218	n=218	n=218
SNP Main Effect	-0.034, 0.789	-0.206, 0.092	0.094, 0.271	-0.004, 0.969	0.051, 0.664	0.138, 0.146	0.028, 0.756	0.056, 0.601
Interaction Model								
SNP Main Effect	0.094, 0.691	-0.233, 0.298	0.247, 0.116	0.18, 0.326	0.129, 0.545	0.258, 0.137	0.279, 0.086	0.244, 0.212
SNP-by-GroupA Inter	-0.188, 0.582	-0.064, 0.844	-0.268, 0.235	-0.098, 0.707	0.212, 0.49	-0.05, 0.843	-0.305, 0.192	-0.25, 0.373
SNP-by-GroupB Inter	-0.181, 0.561	0.114, 0.701	-0.182, 0.379	-0.38, 0.115	-0.352, 0.214	-0.263, 0.252	-0.401, 0.063	-0.284, 0.272
ERCC2rs50872, TT+CT vs. CC								
Main Effects Model	n=214	n=214	n=215	n=214	n=215	n=215	n=215	n=215

SNP Main Effect	0.051, 0.676	0.144, 0.216	0.01, 0.899	0.081, 0.385	0.121, 0.271	0.095, 0.288	0.023, 0.785	0.161, 0.109
Interaction Model								
SNP Main Effect	0.085, 0.673	0.618, 0.001*	0.087, 0.512	0.092, 0.551	0.164, 0.367	0.147, 0.316	0.047, 0.732	0.061, 0.71
SNP-by-GroupA Inter	-0.01, 0.974	-0.561, 0.054	-0.153, 0.458	-0.067, 0.78	0.129, 0.648	0, 1	0.038, 0.86	0.362, 0.158
SNP-by-GroupB Inter	-0.083, 0.769	-0.882, 0.001*	-0.101, 0.593	0.016, 0.942	-0.202, 0.433	-0.141, 0.497	-0.091, 0.641	0.018, 0.939
ERCC3rs2134794, CC+AC vs. AA								
Main Effects Model	n=218	n=218	n=219	n=218	n=219	n=219	n=219	n=219
SNP Main Effect	-0.309, 0.01*	0.169, 0.143	-0.035, 0.666	-0.173, 0.061	0.021, 0.846	-0.05, 0.579	-0.078, 0.351	-0.149, 0.135
Interaction Model								
SNP Main Effect	-0.193, 0.315	0.375, 0.045*	-0.184, 0.157	-0.103, 0.494	0.044, 0.808	0.039, 0.79	-0.182, 0.178	-0.119, 0.46
SNP-by-GroupA Inter	0.05, 0.87	-0.228, 0.442	0.47, 0.023*	0.04, 0.866	-0.088, 0.757	0.012, 0.958	0.375, 0.081	0.225, 0.38
SNP-by-GroupB Inter	-0.346, 0.202	-0.4, 0.128	0.079, 0.666	-0.216, 0.305	0.001, 0.998	-0.247, 0.228	0.023, 0.905	-0.236, 0.3
ERCC3rs4150402, AA+GA vs. GG								
Main Effects Model	n=217	n=217	n=218	n=217	n=218	n=218	n=218	n=218
SNP Main Effect	-0.035, 0.767	-0.216, 0.057	-0.019, 0.811	-0.036, 0.694	-0.033, 0.759	0.017, 0.851	0.09, 0.274	-0.032, 0.745
Interaction Model								
SNP Main Effect	0.045, 0.818	-0.12, 0.516	-0.051, 0.695	-0.167, 0.264	-0.028, 0.873	0.071, 0.625	0.203, 0.131	0.016, 0.923
SNP-by-GroupA Inter	-0.207, 0.503	-0.091, 0.758	0.132, 0.521	0.277, 0.241	0.11, 0.698	-0.153, 0.505	-0.165, 0.439	-0.214, 0.404
SNP-by-GroupB Inter	-0.077, 0.78	-0.197, 0.449	-0.003, 0.989	0.165, 0.434	-0.086, 0.732	-0.042, 0.836	-0.194, 0.308	0.015, 0.947
ERCC3rs4150407, GG+AG vs. AA								
Main Effects Model	n=218	n=218	n=219	n=218	n=219	n=219	n=219	n=219
SNP Main Effect	0.245, 0.052	0.2, 0.1	0.03, 0.723	0.234, 0.016*	0.006, 0.961	-0.036, 0.703	0.031, 0.728	0.073, 0.492
Interaction Model								
SNP Main Effect	0.186, 0.371	0.031, 0.874	0.197, 0.157	0.357, 0.025*	-0.047, 0.808	0.065, 0.676	0.083, 0.569	0.082, 0.683
SNP-by-GroupA Inter	0.089, 0.786	-0.207, 0.511	-0.466, 0.035*	-0.277, 0.269	-0.076, 0.803	-0.106, 0.666	-0.166, 0.471	0.162, 0.557
SNP-by-GroupB Inter	0.097, 0.738	0.546, 0.047*	-0.142, 0.464	-0.145, 0.511	0.18, 0.503	-0.193, 0.374	-0.032, 0.876	-0.122, 0.615
ERCC3rs4150477, TT+CT vs. CC								
Main Effects Model	n=218	n=218	n=219	n=218	n=219	n=219	n=219	n=219

SNP Main Effect	0.111, 0.354	0.094, 0.416	0.045, 0.573	0.19, 0.038*	0.037, 0.735	0.002, 0.982	0.132, 0.112	0.065, 0.514
Interaction Model								
SNP Main Effect	-0.052, 0.79	-0.053, 0.776	0.216, 0.099	0.321, 0.032*	0.005, 0.978	-0.028, 0.849	0.26, 0.056	-0.003, 0.988
SNP-by-GroupA Inter	0.383, 0.222	-0.112, 0.709	-0.417, 0.046*	-0.316, 0.184	-0.185, 0.519	0.064, 0.783	-0.286, 0.186	0.236, 0.366
SNP-by-GroupB Inter	0.188, 0.498	0.453, 0.086	-0.183, 0.323	-0.143, 0.497	0.204, 0.424	0.038, 0.855	-0.154, 0.423	0.029, 0.902
ERCC5rs11069498, GG+AG vs. AA								
Main Effects Model	n=215	n=215	n=216	n=215	n=216	n=216	n=216	n=216
SNP Main Effect	0.085, 0.508	0.002, 0.988	-0.03, 0.726	0.008, 0.936	-0.236, 0.044*	0.065, 0.495	0.02, 0.826	-0.074, 0.488
Interaction Model								
SNP Main Effect	-0.073, 0.723	0.091, 0.648	-0.134, 0.333	0.054, 0.736	-0.38, 0.044*	-0.129, 0.399	-0.2, 0.162	-0.348, 0.043*
SNP-by-GroupA Inter	0.095, 0.782	-0.232, 0.48	0.241, 0.293	-0.331, 0.207	0.479, 0.124	0.536, 0.034*	0.307, 0.194	0.629, 0.027*
SNP-by-GroupB Inter	0.353, 0.229	-0.095, 0.736	0.129, 0.51	0.074, 0.742	0.093, 0.726	0.19, 0.38	0.389, 0.056	0.339, 0.162
ERCC5rs2296147, CC+TC vs. TT								
Main Effects Model	n=216	n=216	n=217	n=216	n=217	n=217	n=217	n=217
SNP Main Effect	0.066, 0.615	0.05, 0.687	0.024, 0.784	-0.054, 0.586	0.049, 0.682	0.008, 0.929	-0.01, 0.915	0.003, 0.981
Interaction Model								
SNP Main Effect	-0.01, 0.964	-0.357, 0.102	-0.211, 0.164	0.003, 0.984	0.178, 0.399	-0.124, 0.463	0.129, 0.417	-0.279, 0.144
SNP-by-GroupA Inter	0.404, 0.237	0.614, 0.058	0.477, 0.034*	-0.137, 0.601	-0.172, 0.583	0.236, 0.345	-0.157, 0.504	0.383, 0.175
SNP-by-GroupB Inter	-0.093, 0.758	0.585, 0.043*	0.249, 0.217	-0.047, 0.842	-0.203, 0.47	0.164, 0.465	-0.237, 0.262	0.435, 0.087
ERCC5rs2296148, TT+CT vs. CC								
Main Effects Model	n=206	n=207	n=207	n=206	n=207	n=207	n=207	n=207
SNP Main Effect	0.108, 0.655	0.022, 0.92	0.015, 0.927	0.011, 0.951	-0.205, 0.345	0.054, 0.767	-0.129, 0.442	0.049, 0.808
Interaction Model								
SNP Main Effect	-0.042, 0.904	0.337, 0.294	0.069, 0.77	-0.335, 0.201	-0.72, 0.021*	0.233, 0.379	-0.339, 0.164	-0.097, 0.74
SNP-by-GroupA Inter	0.16, 0.827	-0.932, 0.163	-0.27, 0.581	0.685, 0.209	0.825, 0.202	-0.444, 0.42	0.36, 0.477	-0.007, 0.991
SNP-by-GroupB Inter	0.35, 0.515	-0.468, 0.34	-0.031, 0.93	0.666, 0.097	1.075, 0.024*	-0.304, 0.452	0.428, 0.251	0.411, 0.359
ERCC5rs4150355, TT+CT vs. CC								
Main Effects Model	n=218	n=218	n=219	n=218	n=219	n=219	n=219	n=219

SNP Main Effect	0.026, 0.828	-0.13, 0.257	0.033, 0.682	0.063, 0.496	0.02, 0.854	0.037, 0.675	0.064, 0.44	0.053, 0.597
Interaction Model								
SNP Main Effect	-0.001, 0.995	-0.298, 0.117	-0.031, 0.814	0.259, 0.091	0.231, 0.206	-0.095, 0.517	0.223, 0.107	-0.151, 0.36
SNP-by-GroupA Inter	0.284, 0.361	0.444, 0.135	0.019, 0.926	-0.387, 0.103	-0.438, 0.123	0.002, 0.993	-0.322, 0.135	0.126, 0.622
SNP-by-GroupB Inter	-0.121, 0.661	0.14, 0.596	0.156, 0.4	-0.249, 0.241	-0.253, 0.318	0.349, 0.089	-0.196, 0.307	0.451, 0.05*
ERCC5rs4150360, CC+TC vs. TT								
Main Effects Model	n=218	n=218	n=219	n=218	n=219	n=219	n=219	n=219
SNP Main Effect	0.133, 0.318	-0.058, 0.648	0.036, 0.69	0.045, 0.662	-0.157, 0.201	0.097, 0.329	0.052, 0.578	0.007, 0.95
Interaction Model								
SNP Main Effect	-0.104, 0.631	0.048, 0.816	-0.01, 0.943	-0.009, 0.959	-0.323, 0.103	-0.074, 0.645	-0.151, 0.317	-0.276, 0.124
SNP-by-GroupA Inter	0.213, 0.55	-0.269, 0.434	0.105, 0.66	-0.096, 0.727	0.508, 0.12	0.568, 0.031*	0.291, 0.24	0.673, 0.023*
SNP-by-GroupB Inter	0.486, 0.111	-0.118, 0.688	0.057, 0.782	0.194, 0.408	0.131, 0.639	0.104, 0.644	0.35, 0.099	0.334, 0.186
ERCC5rs4771436, GG+TG vs. TT								
Main Effects Model	n=215	n=215	n=216	n=215	n=216	n=216	n=216	n=216
SNP Main Effect	0.085, 0.487	-0.083, 0.479	0.039, 0.629	-0.041, 0.663	-0.141, 0.211	0.155, 0.081	0.05, 0.557	0.139, 0.171
Interaction Model								
SNP Main Effect	-0.156, 0.435	0.164, 0.395	-0.055, 0.682	0.022, 0.888	-0.001, 0.994	0.043, 0.771	-0.024, 0.863	0.107, 0.524
SNP-by-GroupA Inter	0.251, 0.426	-0.347, 0.253	0.273, 0.195	-0.257, 0.289	-0.054, 0.852	0.325, 0.157	0.179, 0.414	0.119, 0.651
SNP-by-GroupB Inter	0.465, 0.094	-0.417, 0.119	0.065, 0.726	0.008, 0.969	-0.33, 0.2	0.078, 0.7	0.074, 0.702	0.004, 0.986
ERCC5rs751402, TT+CT vs. CC								
Main Effects Model	n=216	n=216	n=217	n=216	n=217	n=217	n=217	n=217
SNP Main Effect	0.116, 0.349	-0.052, 0.665	0.107, 0.197	-0.081, 0.399	-0.224, 0.05*	0.149, 0.105	-0.04, 0.647	0.146, 0.158
Interaction Model								
SNP Main Effect	-0.008, 0.968	0.246, 0.21	0.075, 0.586	-0.091, 0.57	-0.356, 0.058	-0.075, 0.619	-0.274, 0.054	0.11, 0.519
SNP-by-GroupA Inter	0.105, 0.742	-0.403, 0.186	0.13, 0.539	-0.067, 0.786	0.417, 0.151	0.486, 0.038*	0.499, 0.023*	0.234, 0.377
SNP-by-GroupB Inter	0.261, 0.364	-0.516, 0.061	-0.007, 0.97	0.076, 0.733	0.057, 0.828	0.255, 0.227	0.273, 0.169	-0.072, 0.762
ERCC5rs873601, GG+AG vs. AA								
Main Effects Model	n=214	n=214	n=215	n=214	n=215	n=215	n=215	n=215

SNP Main Effect	-0.288, 0.015*	0.044, 0.704	-0.13, 0.103	0.026, 0.777	-0.227, 0.037*	-0.117, 0.183	-0.154, 0.06	-0.279, 0.005*
Interaction Model								
SNP Main Effect	-0.279, 0.147	0.063, 0.737	-0.077, 0.555	0.036, 0.808	-0.453, 0.011*	-0.143, 0.32	-0.252, 0.059	-0.346, 0.032*
SNP-by-GroupA Inter	0.028, 0.929	0.122, 0.689	-0.191, 0.358	0.09, 0.709	0.309, 0.275	-0.006, 0.98	0.201, 0.348	0.182, 0.481
SNP-by-GroupB Inter	-0.044, 0.872	-0.131, 0.622	-0.016, 0.931	-0.088, 0.677	0.402, 0.107	0.073, 0.72	0.129, 0.493	0.058, 0.797
GPX1rs1050450, GG+AG vs. AA								
Main Effects Model	n=206	n=206	n=207	n=206	n=207	n=207	n=207	n=207
SNP Main Effect	0.009, 0.944	0.167, 0.155	-0.049, 0.54	0.098, 0.298	-0.142, 0.214	-0.004, 0.964	0.04, 0.647	-0.098, 0.336
Interaction Model								
SNP Main Effect	0.139, 0.495	0.136, 0.488	-0.009, 0.949	0.147, 0.353	-0.194, 0.304	-0.078, 0.607	-0.238, 0.093	-0.227, 0.179
SNP-by-GroupA Inter	-0.289, 0.378	0.025, 0.938	-0.028, 0.895	-0.06, 0.81	0.464, 0.124	0.202, 0.406	0.298, 0.187	0.113, 0.675
SNP-by-GroupB Inter	-0.158, 0.572	0.063, 0.812	-0.083, 0.649	-0.084, 0.697	-0.135, 0.599	0.067, 0.746	0.509, 0.009*	0.252, 0.275
PARP1rs1136410, CC+TC vs. TT								
Main Effects Model	n=216	n=216	n=217	n=216	n=217	n=217	n=217	n=217
SNP Main Effect	0.105, 0.434	-0.065, 0.61	0.04, 0.652	-0.104, 0.309	-0.001, 0.992	0.067, 0.499	0.075, 0.422	0.084, 0.454
Interaction Model								
SNP Main Effect	0.256, 0.269	-0.244, 0.274	0.037, 0.81	-0.107, 0.548	-0.002, 0.992	-0.017, 0.919	0.11, 0.495	0.135, 0.483
SNP-by-GroupA Inter	-0.38, 0.281	0.266, 0.432	0.119, 0.614	-0.133, 0.623	-0.01, 0.974	0.312, 0.233	-0.003, 0.989	0.199, 0.498
SNP-by-GroupB Inter	-0.138, 0.659	0.271, 0.368	-0.064, 0.759	0.087, 0.718	0.009, 0.976	0.016, 0.945	-0.083, 0.702	-0.244, 0.35
PARP1rs2271347, AA+GA vs. GG								
Main Effects Model	n=216	n=216	n=217	n=216	n=217	n=217	n=217	n=217
SNP Main Effect	0.182, 0.142	0.119, 0.32	0.183, 0.027*	0.202, 0.034*	0.151, 0.188	0.013, 0.888	0.109, 0.207	0.076, 0.465
Interaction Model								
SNP Main Effect	0.102, 0.601	-0.005, 0.9	0.502, <0.001*	0.328, 0.029*	0.315, 0.08	-0.026, 0.86	0.196, 0.148	0.404, 0.013*
SNP-by-GroupA Inter	0.077, 0.814	-0.187, 0.547	-0.589, 0.006*	-0.245, 0.325	-0.38, 0.205	-0.089, 0.713	-0.368, 0.104	-0.596, 0.027*
SNP-by-GroupB Inter	0.169, 0.544	0.445, 0.095	-0.498, 0.006*	-0.191, 0.371	-0.211, 0.41	0.158, 0.448	-0.011, 0.953	-0.519, 0.025*
PARP1rs3219058, AA+GA vs. GG								
Main Effects Model	n=218	n=218	n=219	n=218	n=219	n=219	n=219	n=219

SNP Main Effect	0.006, 0.963	0.028, 0.82	-0.173, 0.042*	-0.053, 0.585	0.036, 0.759	0.036, 0.701	-0.133, 0.133	0.097, 0.362
Interaction Model								
SNP Main Effect	-0.093, 0.642	-0.036, 0.85	-0.243, 0.069	-0.1, 0.515	0.011, 0.954	0.012, 0.937	-0.152, 0.274	0.09, 0.59
SNP-by-GroupA Inter	0.397, 0.235	0.06, 0.848	0.088, 0.687	0.18, 0.475	0.311, 0.303	-0.035, 0.887	0.17, 0.456	0.097, 0.724
SNP-by-GroupB Inter	0.016, 0.957	0.143, 0.617	0.139, 0.483	0.008, 0.972	-0.147, 0.59	0.096, 0.665	-0.065, 0.752	-0.049, 0.843
PARP1rs3219090, AA+GA vs. GG								
Main Effects Model	n=192	n=192	n=193	n=193	n=193	n=193	n=193	n=193
SNP Main Effect	0.092, 0.477	-0.016, 0.893	-0.009, 0.916	-0.079, 0.416	0.013, 0.91	0.059, 0.54	-0.034, 0.686	0.18, 0.094
Interaction Model								
SNP Main Effect	0.037, 0.866	-0.097, 0.64	-0.072, 0.611	-0.094, 0.564	-0.105, 0.598	-0.016, 0.922	-0.148, 0.305	0.295, 0.102
SNP-by-GroupA Inter	-0.027, 0.936	-0.059, 0.857	0.149, 0.499	-0.068, 0.789	0.267, 0.392	0.159, 0.531	0.189, 0.399	0.038, 0.893
SNP-by-GroupB Inter	0.156, 0.596	0.239, 0.395	0.063, 0.742	0.084, 0.706	0.128, 0.637	0.086, 0.695	0.164, 0.4	-0.316, 0.197
SEPP1rs230819, AA+CA vs. CC								
Main Effects Model	n=211	n=211	n=212	n=211	n=212	n=212	n=212	n=212
SNP Main Effect	0.154, 0.273	-0.007, 0.956	0.069, 0.466	0.255, 0.018*	-0.134, 0.307	0.103, 0.322	0.103, 0.298	-0.008, 0.949
Interaction Model								
SNP Main Effect	0.13, 0.586	0.05, 0.827	0.113, 0.484	0.199, 0.278	0.048, 0.831	0.053, 0.763	0.001, 0.996	-0.017, 0.931
SNP-by-GroupA Inter	0.084, 0.828	0.02, 0.957	-0.093, 0.715	0.07, 0.809	-0.198, 0.576	0.059, 0.833	0.047, 0.86	0.003, 0.993
SNP-by-GroupB Inter	0.016, 0.959	-0.139, 0.652	-0.054, 0.8	0.093, 0.703	-0.316, 0.289	0.083, 0.725	0.209, 0.352	0.021, 0.938
SEPP1rs28919892, CC+TC vs. TT								
Main Effects Model	n=216	n=216	n=217	n=216	n=217	n=217	n=217	n=217
SNP Main Effect	0.18, 0.134	0.118, 0.307	0.07, 0.377	0.143, 0.119	0.002, 0.989	0.012, 0.89	0.072, 0.39	-0.083, 0.409
Interaction Model								
SNP Main Effect	-0.045, 0.818	0.006, 0.974	0.087, 0.504	-0.102, 0.493	-0.052, 0.771	-0.063, 0.667	0.01, 0.943	-0.205, 0.207
SNP-by-GroupA Inter	0.532, 0.087	0.063, 0.834	-0.017, 0.933	0.391, 0.098	0.285, 0.319	0.218, 0.347	0.088, 0.685	0.428, 0.098
SNP-by-GroupB Inter	0.249, 0.364	0.257, 0.333	-0.033, 0.857	0.394, 0.062	-0.047, 0.853	0.055, 0.789	0.107, 0.578	0.041, 0.857
SEPP1rs3877899, GG+AG vs AA								
Main Effects Model	n=209	n=209	n=210	n=209	n=210	n=210	n=210	n=210

SNP Main Effect	-0.123, 0.333	-0.182, 0.132	-0.07, 0.408	-0.047, 0.623	-0.327, 0.005*	-0.037, 0.69	-0.085, 0.329	-0.101, 0.336
Interaction Model								
SNP Main Effect	0.043, 0.836	0.079, 0.69	-0.044, 0.751	0.273, 0.084	-0.149, 0.426	0.024, 0.878	-0.094, 0.517	-0.092, 0.598
SNP-by-GroupA Inter	-0.51, 0.119	-0.432, 0.166	-0.248, 0.251	-0.538, 0.029*	-0.571, 0.051	-0.351, 0.144	0.021, 0.926	-0.208, 0.441
SNP-by-GroupB Inter	-0.099, 0.729	-0.395, 0.149	0.097, 0.612	-0.474, 0.03*	-0.085, 0.741	0.073, 0.731	0.009, 0.965	0.112, 0.639
SOD1rs1041740, TT+CT vs. CC								
Main Effects Model	n=213	n=213	n=214	n=213	n=214	n=214	n=214	n=214
SNP Main Effect	0.108, 0.363	0.09, 0.441	-0.007, 0.927	0.254, 0.006*	0.07, 0.53	-0.105, 0.239	0.085, 0.314	0.112, 0.271
Interaction Model								
SNP Main Effect	0.001, 0.997	-0.204, 0.28	0.142, 0.282	0.245, 0.104	0.37, 0.041*	-0.029, 0.844	0.174, 0.205	0.095, 0.565
SNP-by-GroupA Inter	0.168, 0.603	0.48, 0.131	-0.125, 0.568	-0.104, 0.676	-0.244, 0.414	-0.088, 0.715	-0.166, 0.466	-0.067, 0.808
SNP-by-GroupB Inter	0.177, 0.516	0.475, 0.072	-0.305, 0.098	0.082, 0.696	-0.619, 0.015*	-0.143, 0.482	-0.131, 0.496	0.079, 0.732
SOD2rs4880, TT+CT vs. CC								
Main Effects Model	n=213	n=213	n=214	n=214	n=214	n=214	n=214	n=214
SNP Main Effect	0.017, 0.903	-0.303, 0.024*	0.004, 0.97	0.097, 0.364	-0.03, 0.815	-0.077, 0.456	0.042, 0.661	0.11, 0.349
Interaction Model								
SNP Main Effect	0.117, 0.623	-0.427, 0.06	-0.071, 0.657	0.285, 0.12	-0.157, 0.478	-0.033, 0.85	0.006, 0.972	0.035, 0.861
SNP-by-GroupA Inter	-0.027, 0.945	0.536, 0.14	0.174, 0.497	-0.258, 0.378	0.159, 0.652	-0.229, 0.416	0.047, 0.859	-0.123, 0.701
SNP-by-GroupB Inter	-0.219, 0.5	0.009, 0.978	0.084, 0.699	-0.306, 0.218	0.215, 0.474	0.018, 0.94	0.062, 0.783	0.242, 0.373
SOD2rs5746136, AA+GA vs. GG								
Main Effects Model	n=217	n=217	n=218	n=217	n=218	n=218	n=218	n=218
SNP Main Effect	-0.018, 0.881	-0.257, 0.023*	0.099, 0.21	-0.027, 0.765	0.012, 0.915	-0.06, 0.49	0.096, 0.238	0.062, 0.531
Interaction Model								
SNP Main Effect	-0.009, 0.963	-0.325, 0.082	0.102, 0.44	-0.13, 0.388	-0.128, 0.479	-0.102, 0.483	0.138, 0.314	-0.142, 0.383
SNP-by-GroupA Inter	-0.027, 0.929	0.138, 0.64	-0.015, 0.94	0.365, 0.12	0.385, 0.174	0.062, 0.784	-0.129, 0.544	0.169, 0.507
SNP-by-GroupB Inter	-0.004, 0.988	0.089, 0.733	0.002, 0.989	0.024, 0.908	0.108, 0.67	0.069, 0.736	-0.021, 0.911	0.421, 0.065
SOD2rs8031, TT+AT vs. AA								
Main Effects Model	n=218	n=218	n=219	n=218	n=219	n=219	n=219	n=219

SNP Main Effect	-0.036, 0.794	-0.332, 0.011*	0.004, 0.963	0.045, 0.668	-0.046, 0.717	-0.108, 0.294	-0.011, 0.912	0.092, 0.423
Interaction Model								
SNP Main Effect	0.069, 0.766	-0.379, 0.083	-0.044, 0.78	0.253, 0.156	-0.182, 0.394	-0.066, 0.705	-0.016, 0.92	0.007, 0.971
SNP-by-GroupA Inter	0.008, 0.982	0.5, 0.15	0.205, 0.41	-0.206, 0.468	0.173, 0.611	-0.196, 0.475	0.132, 0.609	-0.09, 0.77
SNP-by-GroupB Inter	-0.26, 0.417	-0.171, 0.568	-0.001, 0.998	-0.393, 0.11	0.236, 0.422	0.01, 0.967	-0.062, 0.781	0.262, 0.326

Note. *=p<.05. [a]=Reported b-coefficients are from regression models with modified influential point values. Group A=prescribed chemotherapy plus anastrozole; Group B=prescribed anastrozole alone; SNP=Single Nucleotide Polymorphism; Inter=Interaction.
