# Supplementary material for: Polymorphisms in DNA repair and oxidative stress genes associated with pre-treatment cognitive function in breast cancer survivors: an exploratory study
Source: Springerplus. 2016 Apr 9;5:422. doi: 10.1186/s40064-016-2061-4 (PMC4826652; doi:10.1186/s40064-016-2061-4)
Supplement: Supplementary file 2 — 10.1186/s40064-016-2061-4 Oxidative stress and DNA repair gene-gene networks generated by pathway analysis. [file 40064_2016_2061_MOESM2_ESM.pdf]

Polymorphisms in DNA repair and oxidative stress genes associated with pre-treatment cognitive function in breast cancer survivors: an exploratory study

Breast Cancer Research and Treatment

Theresa A. Koleck[1] (tat30@pitt.edu), Catherine M. Bender[1], Susan M. Sereika[1,2], Adam M. Brufsky[3-5], Barry C. Lembersky[3,4], Priscilla F. McAuliffe[5,6], Shannon L. Puhalla[3,5], Priya Rastogi[3,5] & Yvette P. Conley[1,7]

[1]School of Nursing, University of Pittsburgh, 3500 Victoria Street, Pittsburgh, PA 15261, USA
[2]Department of Biostatistics and Department of Epidemiology, Graduate School of Public Health, University of Pittsburgh, 130 De Soto Street, Pittsburgh, PA 15261, USA
[3]Division of Hematology/Oncology, Magee-Womens Hospital of University of Pittsburgh Medical Center (UPMC), 300 Halket Street, Pittsburgh, PA 15213, USA
[4]University of Pittsburgh Cancer Institute, 5150 Centre Avenue, Pittsburgh, PA 15232, USA
[5]School of Medicine, University of Pittsburgh, 3550 Terrace Street, Pittsburgh, PA 15261, USA
[6]Magee-Womens Surgical Associates, Magee-Womens Hospital of University of Pittsburgh Medical Center (UPMC), 300 Halket Street, Pittsburgh, PA 15213, USA
[7]Department of Human Genetics, Graduate School of Public Health, University of Pittsburgh, 130 De Soto Street, Pittsburgh, PA 15261, USA

Network 1

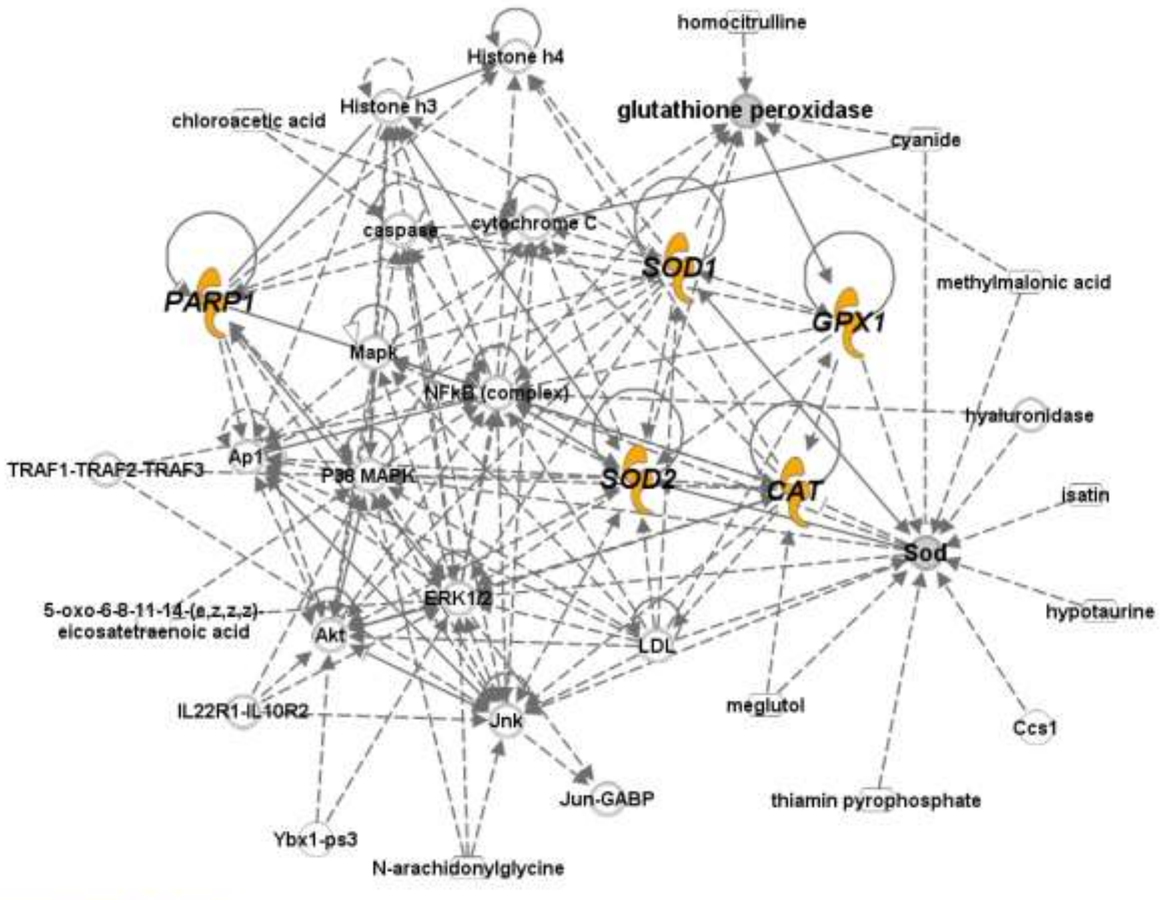

© 2000-2016 QIAGEN. All rights reserved.

Network 2

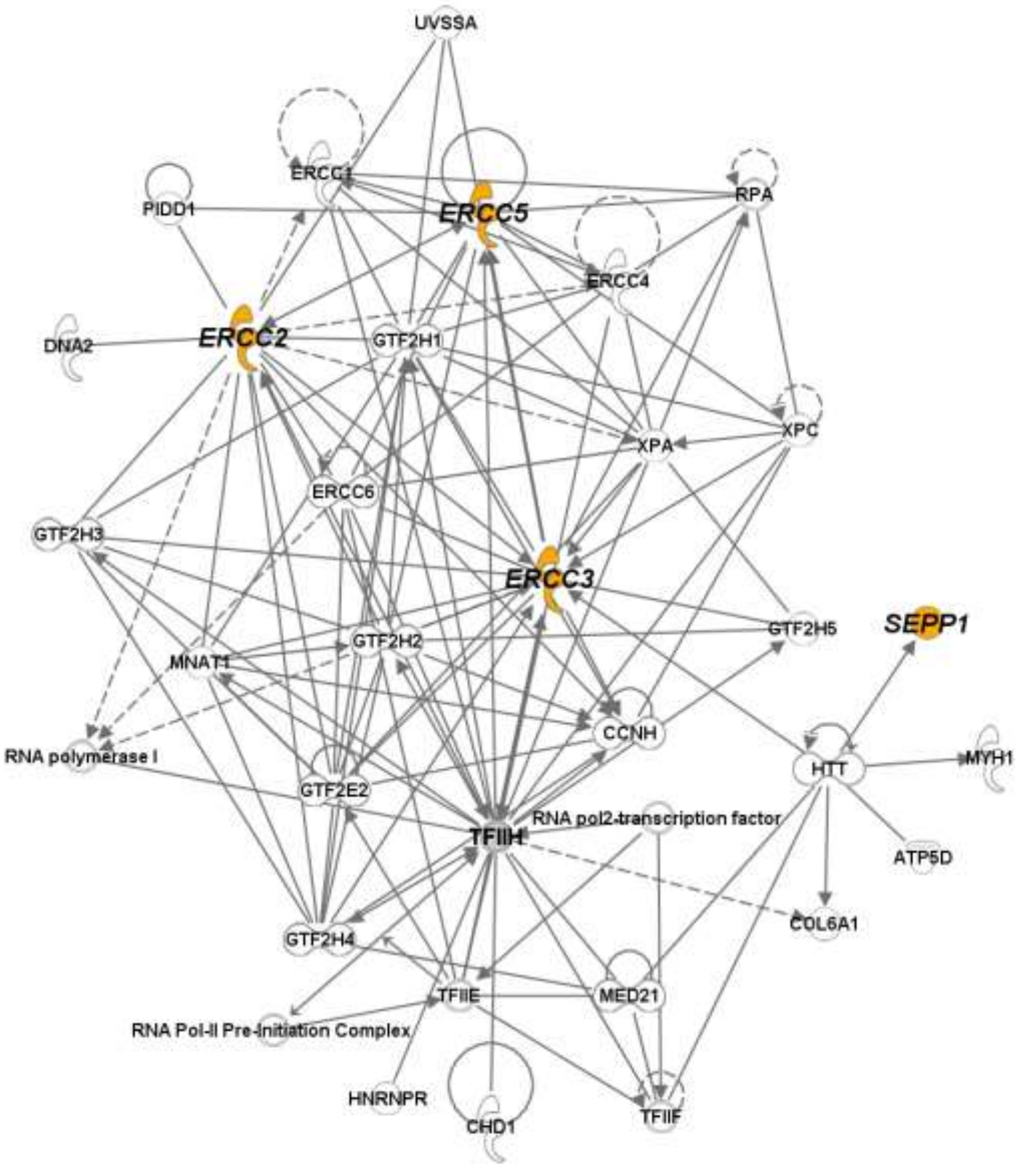

© 2000-2016 QIAGEN. All rights reserved.

Online Resource 2 Oxidative stress and DNA repair gene-gene networks generated by pathway analysis

Note. The networks were generated through the use of QIAGEN's Ingenuity Pathway Analysis (IPA®, QIAGEN Redwood City, www.qiagen.com/ingenuity). The main associated functions of each network are: (Network 1) free radical scavenging, small molecule biochemistry, and neurological disease and (Network 2) DNA replication, recombination, and repair, energy production, and nucleic acid metabolism.
